# Sulfamethoxazole Induces a Switch Mechanism in T Cell Receptors Containing TCRVβ20-1, Altering pHLA Recognition

**DOI:** 10.1371/journal.pone.0076211

**Published:** 2013-10-07

**Authors:** Stephan Watkins, Werner J. Pichler

**Affiliations:** 1 Department of Rheumatology, Clinical Immunology and Allergology, Inselspital/University Hospital of Bern, Bern, Switzerland; 2 Department of Graduate Cell Biology, University of Bern, Bern, Switzerland; German Cancer Research Center, Germany

## Abstract

T cell receptors (TCR) containing Vβ20-1 have been implicated in a wide range of T cell mediated disease and allergic reactions, making it a target for understanding these. Mechanics of T cell receptors are largely unexplained by static structures available from x-ray crystallographic studies. A small number of molecular dynamic simulations have been conducted on TCR, however are currently lacking either portions of the receptor or explanations for differences between binding and non-binding TCR recognition of respective peptide-HLA. We performed molecular dynamic simulations of a TCR containing variable domain Vβ20-1, sequenced from drug responsive T cells. These were initially from a patient showing maculopapular eruptions in response to the sulfanilamide-antibiotic sulfamethoxazole (SMX). The CDR2β domain of this TCR was found to dock SMX with high affinity. Using this compound as a perturbation, overall mechanisms involved in responses mediated by this receptor were explored, showing a chemical action on the TCR free from HLA or peptide interaction. Our simulations show two completely separate modes of binding cognate peptide-HLA complexes, with an increased affinity induced by SMX bound to the Vβ20-1. Overall binding of the TCR is mediated through a primary recognition by either the variable β or α domain, and a switch in recognition within these across TCR loops contacting the peptide and HLA occurs when SMX is present in the CDR2β loop. Large binding affinity differences are induced by summed small amino acid changes primarily by SMX modifying only three critical CDR2β loop amino acid positions. These residues, TYRβ57, ASPβ64, and LYSβ65 initially hold hydrogen bonds from the CDR2β to adjacent CDR loops. Effects from SMX binding are amplified and traverse longer distances through internal TCR hydrogen bonding networks, controlling the overall TCR conformation. Thus, the CDR2β of Vβ20-1 acts as a ligand controlled switch affecting overall TCR binding affinity.

## Introduction

Research on T cell (TC) mediated adverse drug and hypersensitivity to small molecule pharmaceuticals has traditionally been focused on interactions with peptide-human leukocyte antigen (pHLA) [1,2]. The TC receptor (TCR) is often shown to be restricted to interactions involving recognition of these molecules as presented in stable forms, such as haptenized peptides, through complement determining region (CDR) recognition of the stabilized molecule-pHLA [3]. Studies of secondary interactions of these compounds with a TCR alone is often complicated by the high level of variability found in the CDR3 regions of the TCR, along with a large repertoire of variable α/β domains expressed in any one individual [4]. Correlations in a number of pharmaceutical induced reactions have been shown for specific TCR subsets [5-7]. These neglected interactions are now receiving more focus, and models developed on possible mechanisms of induced hypersensitivity reactions caused through the TCR alone [8,9].

**Figure 1:**
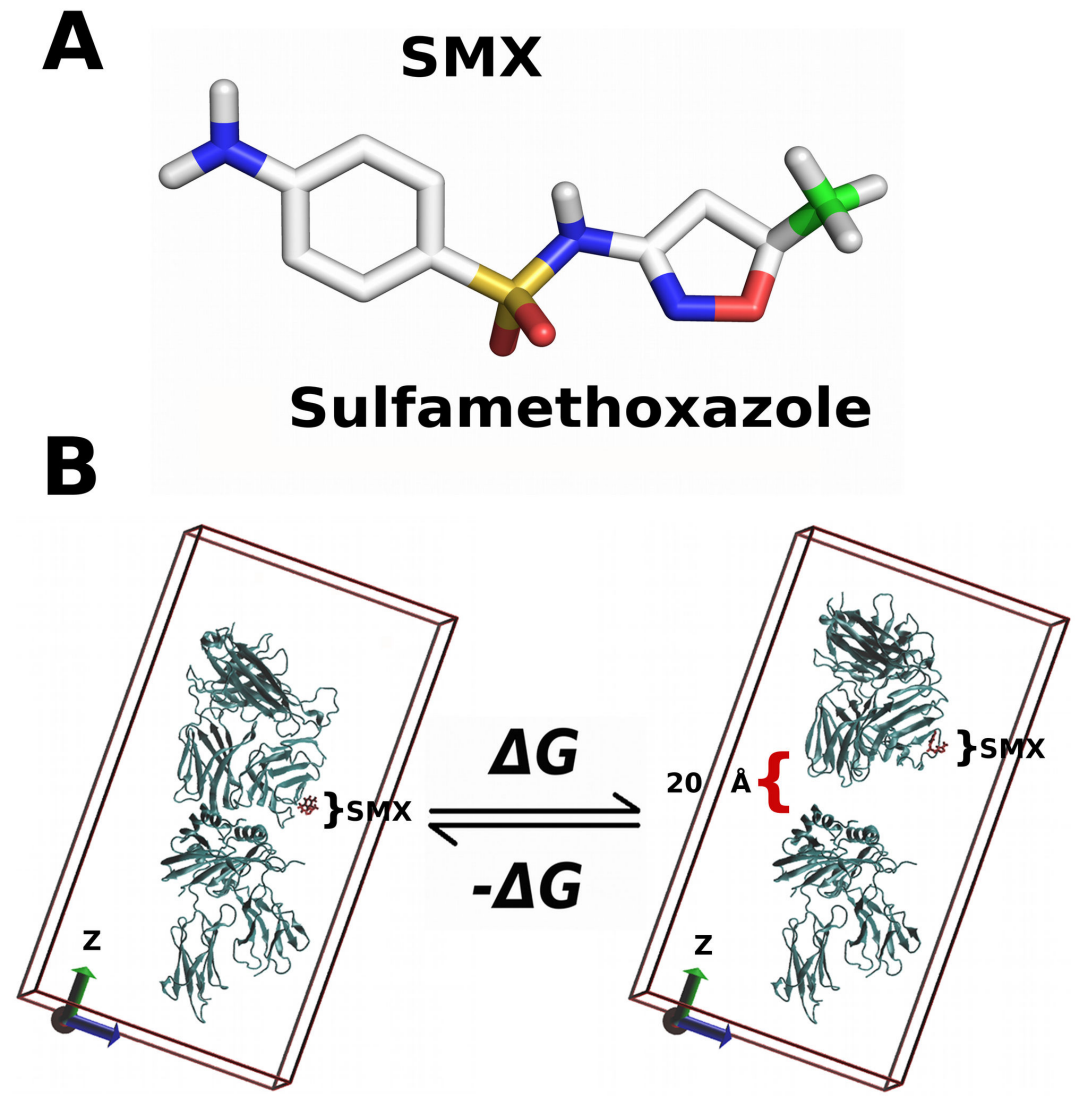
Simulation Design. **A**) Structure of Sulfamethoxazole, used in MD simulations. Green, methyl, yellow, sulfur, red, oxygen, blue, nitrogen, white, other carbon and hydrogen. Aromatic carbon hydrogens are not shown. **B**) Schematic of the MD simulation in initial and final state. ΔG for the reaction is simply the reverse of the simulation path, affinity is taken as the - ΔG. Box represents entire unit cell without water, with Z axis as the long dimension, showing direction pull force was applied in the direction of the arrow. SMX is shown as red stick modeled into CDR2β loop of the TCR in an initial docked, and final position after 4ns as indicated by brackets, pHLA is lower fixed portion of model. The distance moved at the last frame is indicated with black bracket and distance.

Our work focuses on a TC isolated from patients showing maculopapular eruptions from Sulfamethoxazole (SMX), a second resort antibiotic which has high incidents of adverse drug reactions (ADR) and hypersensitivity [10-13]. Due to a wide scope of already determined interactions with SMX ranging from highly affine Ig, pHLA, haptenated serum proteins and some suspected interactions with TCR alone, this compound is ideal for probing most drug interactions. While a number of small molecules show skewing towards various HLA subtypes, such as β-lactam antibiotics or Abacavir, SMX shows no such skewing. Other studies, such as those with Carbamazepine ADR show both skewing of HLA types in some cases, or skewed TCR variable (V) domain (TCRV) α or β in other studies and seem to be HLA dependent [14,15]. A number of studies show other TCRV skewing in different pathologies from allergy or drug induced allergy as well [7,16]. In our preliminary studies, a number of TCR from TC responding to SMX, from SMX induced ADR patients had been sequenced. From docking of SMX to models of these TCR (Figure 1A), we found an interaction with one TCR subtype containing Vβ20-1 and Vα17-1 which had no direct interaction with the peptide or HLA interface.

Varied autoimmune pathologies have been associated with TCR Vβ20-1 skewing as well as skin homing, suggesting some indirect effect in pathology [17]. These analyzed pathologies are primarily mucosa associated, and show a skewed TCR Vα repertoire, Vα7-1 and 17-1 in particular, from TC isolated from salivary glands in Sjögren’s syndrome. Other Vβ20-1 pathologies include chronic IgG mediated allergies, Autoimmune Encephalomyelitis and other papular TC isolates [6,16,18,19]. Additionally, in some cases Vβ20-1 containing TC have also been implicated in tissue rejection, however these remain controversial [20]. For all cases, these TC show a limited degree of isolated natural peptide ligands all of which are associated with basement membrane proteins [19,21,22]. In our case, lamaninα2 has been shown to be an associated ligand, and is particular to overproduction in Sjögren’s syndrome as an indicator of disease [23,24]. This would suggest Vβ20-1 containing α/β TC might be a subset of TC with a natural peptide ligand, or be associated with specific tissues. Indeed, this has been recently shown for Vβ20-1 TCR which were identified as skin homing or associated with nerve tissue [17,19]. However in ADR and disease models, both types of T cell mediated reactions are complicated by several associational factors, such as HLA type, overproduction of a particular protein, or in the case of Ig polyps or Sjögren’s syndrome initial Ig mediated reactions and several other immune cell types [18,24,25]. In all these cases, the chronic inflammatory responses are eventually sustained through T cells. With ADR, induced reactions mimic many disease pathologies also found in autoimmune reactions [26,27].

Based on static models of TCR-pHLA from x-ray data, no apparent effects are directly observable, which might indicate this particular TCR subtype contains a molecule ligand-binding motif or harbors a site that can interact with small molecules free from the pHLA interface [17,28,29]. In addition, x-ray models of TCR-pHLA interactions give conflicting modes of binding and suggest no overall means for signaling in TCR other than simple affinities associated with the protein-protein interactions themselves [30,31]. This has as a consequence led to two different theories of TCR mechanics in general, related to overall conformational changes or simple affinity for a particular peptide as the driving mechanism leading to TCR induced signaling [32-34]. In the first theory, loops facing the pHLA are slightly flexible, and recognition of the proper pHLA alone induces larger TCR conformational changes which are subsequently amplified through attached auxiliary cluster of differentiation (CD) molecules [35,36]. In a second model, the TCR is largely unchanging with a fixed HLA affinity respective of variable domains used, and minor or no movement in the TCR when engaging the pHLA. The hyper-variable CDR3α and β loops, defining the unique amino acids on all TCR, then recognize specific peptide amino acids presented by the HLA. This model simply relies on kinetics related to the overall time a TCR remains attached to the pHLA, working like a simple lock and key. For T cell activation, a TCR-pHLA engagement period beyond a determined time threshold is required to induce Ca2++ signaling. If the affinity of the TCR for the pHLA is met based on either CDR3 loops additional hydrogen bonding added to an already fixed variable domains unchanging affinity, Ca2++ signaling occurs. The large number of additional membrane proteins associated with the entire process further complicates either model.

How small molecules may elicit ADR in either case is often focused on the pHLA based on either theory. In a model where no conformational changes occur in the TCR, small molecules themselves can only alter the overall affinity through binding to the pHLA or TCR at the TCR-pHLA recognition interface, or subsequent protein-protein interface. These would simply alter the kinetics, driving them in one direction or the other and thus altering the normal response. This does not negate other interaction sites than pHLA, such as with the CD3 molecules, where the actual sites of interaction are only partially determined. If the TCR has larger conformational changes, a small molecule itself may also cause associated ADR by binding to sites on the TCR directly, not related to interactions with CD molecules, or the pHLA. Structural studies remain problematic in deciphering TCR induced drug allergies, or mechanics, as themselves represent only super-cooled static representations of single states. Molecular dynamics (MD) has been used to further TCR-pHLA interaction models, however, these have been limited by missing regions of the TCR, such as the constant domains, or did not discern between recognized or non-recognized peptides related to changes in the TCR [37-39].

Here, we use MD of a complete extracellular TCR-pHLA in solvent to explore the effects of a drug ligand already shown to illicit a response in TC, at a molecular level (Figure 1B). Our aim is to show an effect from SMX, and additionally to explore the mechanisms of TCR recognition of the cognate pHLA. This latter is one fundamental aspect often currently cited as a reason TCR mediated ADR are not fully understood. In our model, we focus on a site of small molecule interaction on the TCR free from the pHLA interface. Additionally, we aim to provide explanations of differences found in X-ray structures of TCR-pHLA, applied to TCR binding mechanisms themselves. This TCR was chosen based on the docking of SMX to a novel site not characterized in previous studies found associated with ADR or small molecule interactions. Some crystallographic data exist showing this TCR to be the only interacting subtype with toxic shock syndrome toxin 1 (TSST-1), also binding to the same area as SMX on the TCR [40]. In most of these studies, discrepancies in TCR variable domain nomenclature exist, where Vβ20-1, using the IMGT nomenclature [41], is referred to as Vβ2, Vβ2.1 or Vβ2s1.

Our simulations employ pulled force MD, a type of steered MD, which allows for changes in free energy to be determined within short simulation times. This method also allows conformational changes to be analyzed for proteins not constrained, however the method always constrains one of the proteins involved [37,42-44]. These simulations work by applying an external energy that pulls the center of mass (COM) of one molecule, here the TCR, away from the pHLA. This force is applied over a gradient during the simulation process from 0 to the maximum required to move the TCR away from the pHLA pre-defined by the user which may vary, and is used directly to determine binding affinity between two proteins or molecules. Simulations follow standards developed for protein-ligand dynamics, which also include sampling space over initial starting points defining a Gaussian distribution around mean affinities [45]. These distributions reflect a more realistic natural affinity, as at the molecular level free energy becomes largely defined as a many-bodied summation because of the overall effects of movement and introduced randomized effects of all atoms contributing to calculations. However, as an artificial force is used to speed up simulation times from microseconds to nanoseconds, rate kinetics related to association or dissociation times cannot be determined. This leaves only total free energy, or binding affinity, as the determinant kinetic parameter.

## Results

### Initial Model Generation

**Figure 2:**
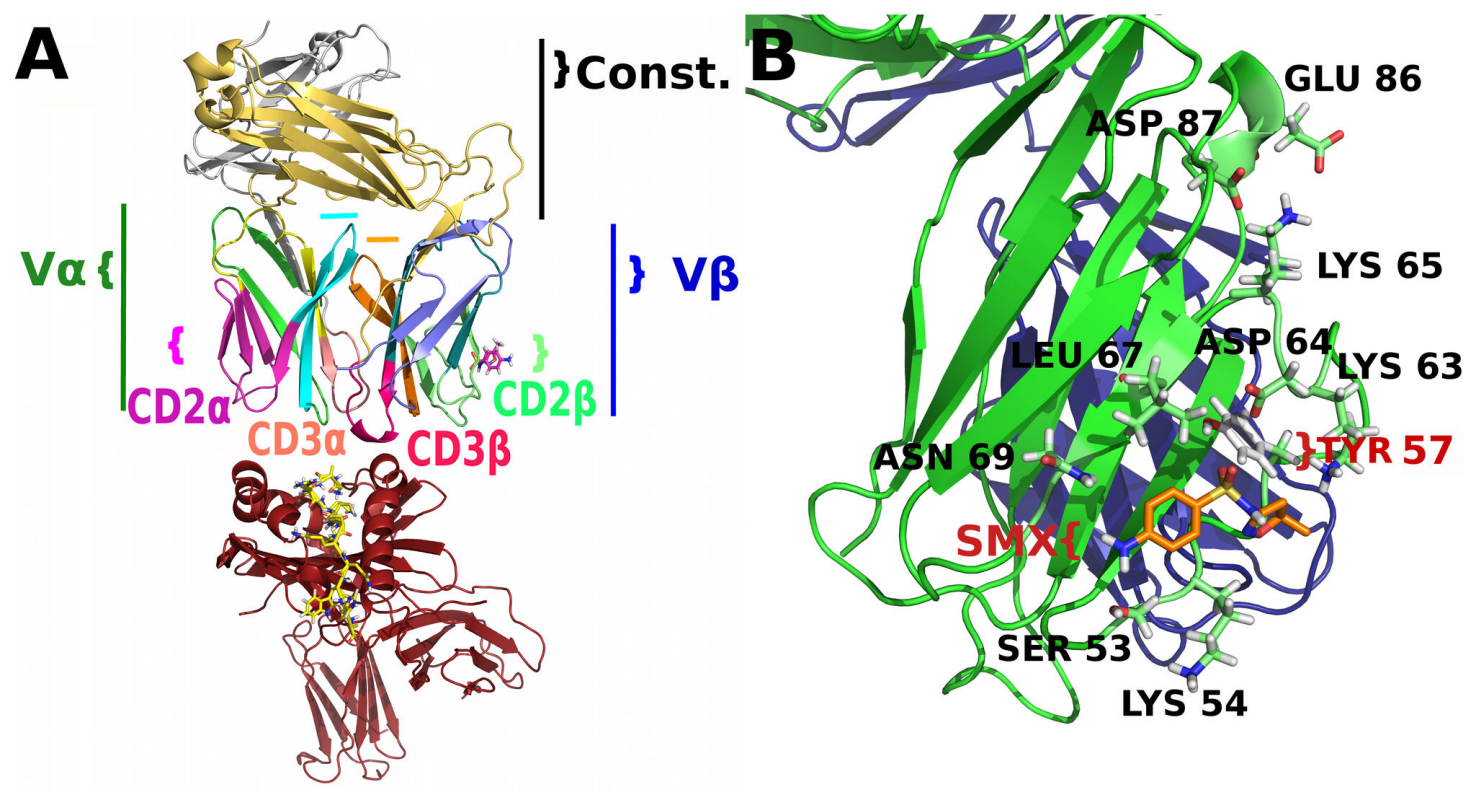
Models Used in Simulation. **A**) Model used in MD simulation, TCR and pHLA shown 20 Å apart. HLA, red, TCR color-coded according to domains used in analysis. Vα/β, variable domain α or β. SMX, stick model on CD2β, present in liganded simulations. Peptide, yellow stick model as bound in HLA. Important TCR domains also have adjacent labels with same colors as domain in model. Const., Constant domain shown as gold, β or grey, α. Colored bar in mid portion of TCR, aqua, CDR1-2α upper connecting loop, orange, CDR1-2β upper connecting loop. TCR is oriented with β domain to right. **B**) Close up of CDR2β, with SMX stick model as Docked. TCR is in bound position to the pHLA from starting models. Labeled residues form portion of hydrogen bonding network affected by SMX and analyzed in detail, or directly hydrogen bonding to SMX. TCRVβ, green, TCRVα, blue.

The model used in this study was originally developed based on series of TC shown to give a proliferative response to SMX. Models of whole TCR were generated using automated modeling web servers as described in methods, as well as the HLA-DRB10. For the Vβ20-1 TCR, several x-ray structures exist which vary only for 4-5 residues at the CDR3 loop, and equivalently the J segment. Inclusive of the x-ray structures for HLA-DRB10 and the TCRVα, this allowed our initial model to be based on 92-93% identical homology with solved structures. For this study, an additional peptide ligand was determined independently from published work, which would suggest basement membrane ligands as possible peptide fragment ligands. Here, Lamaninα2 was found as a high affinity ligand from methodical docking as described, which showed some correlation based on literature for this TCR subtype [23,24]. The entire model was initiated from a SMX bound conformation found through extensive docking analysis, and two starting points for simulations began with an SMX bound or removed point, and were solvated as described (Figure 2). Simply removing SMX, and allowing the TCR-pHLA to equilibrate at 300K in solvent without restraints changed the initial starting conformation contacts between the two proteins. Normal TCR-pHLA recognition occurs in ms to second time frames even for non-recognized pHLA, thus such unrestrained equilibrations of this model system allow TCR in both high or low affinity recognition to be properly modeled as they would occur naturally [38,45].

Based on these equilibrated models, differences were already apparent based on the pHLA contact conformation adopted during equilibration. These included differences between primarily the CDR loops comprising the pHLA contact interface, as seen in the initial frame of all supplemental movies. Additional differences were apparent in the constant domain as it was held, which differed by 5-7Å when superimposed. In addition, the TCRVβ residues β220-230 which contact the J segment directly were held 1Å further away from the J segment with respect to the entire 10 amino acid domain. Overall, there was no apparent difference in hydrogen bonding which would suggest one conformation was properly recognizing the pHLA in a bound conformation. Initial starting models contained roughly 25 for the SMX bound and 28 for the non-bound pHLA interface hydrogen bonds. Based on these models, MD simulations were conducted to discern between differences in the overall pHLA binding process, which would allow for SMX induced changes in one state from the other to become clear.

For CDR2β representing the ligand binding site, initial surface area and volumes were 29.552 (+/- 0.10) nm^2^, 5.76 (+/- 0.06) nm^3^, 28.59 (+/- 0.15) nm^2^ and 5.70 (+/- 0.07) nm^3^ for SMX bound versus free. Using the TCR as whole, area and volume differences were 240.452 (+/- 0.41) nm^2^, 76.54 (+/- 0.214) nm^3^, 247.44 (+/- 0.70) nm^2^ and 77.20 (+/- 0.192) nm^3^ with and without SMX. These are mainly in the solvent accessible surface, however the binding site only represents 14% of this surface difference, while representing only 8-9% of the total volume change observed in the TCR. From starting models, it was apparent long range differences, or structural changes must occur based on binding of SMX alone and these differences are not simple volume changes from occupancy of a particular site.

### Total Energy Between Models

**Figure 3:**
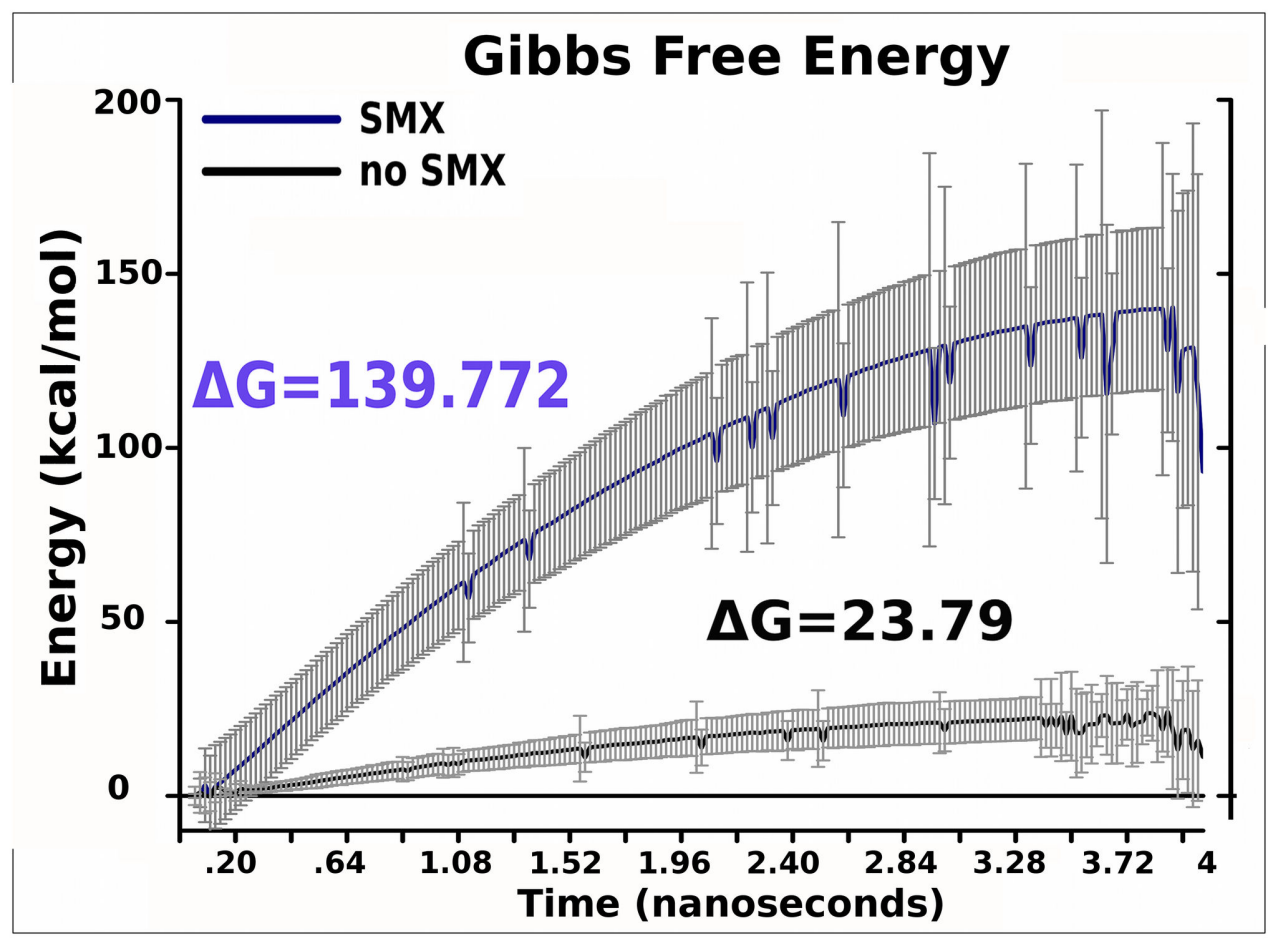
Total Free Energy Change. Free energy calculated from all simulations. Grey bars, standard deviation, Means colored lines, purple SMX, black without SMX, energy is in kcal/mol. Final SHAM calculated free energy changes are indicated on graph as ΔG for the forward reaction, taken at 3.8 ns. The TCR in each simulation set becomes completely disengaged from the pHLA between 3.2-3.4 ns, which is more apparent in simulation paths without SMX.

Total Gibbs free energy changes from SHAM calculated means of the two composite simulations (Figure 3, Figure S1, Movie S1, S2), showed a 7 fold increase in affinity from the TCR alone to the TCR with SMX bound in the CDR2β. These correspond to a change of 2 to 0.79 µmol affinity, that represents a normal pHLA self interaction to an equivalent weak natural killer T cell affinity for a responsive ligand. All represented affinity changes are shown in reverse based on pull simulation analytical methods, with higher energy corresponding to a higher affinity. The pull force is used along with the initial energies of the modeled system in the computational mathematics to determine total energy change, inclusive of solvent. This method however shows only total energy change, and as a result, itself does not allow for energy changes respective of a single domain, residue or subunit to be determined. This analysis may therefore miss changes for a particular component, such as a single loop or domain change, and is used only to determine protein-protein or protein-ligand binding affinities.

### Individual Domain Energy Changes Between Models

**Figure 4:**
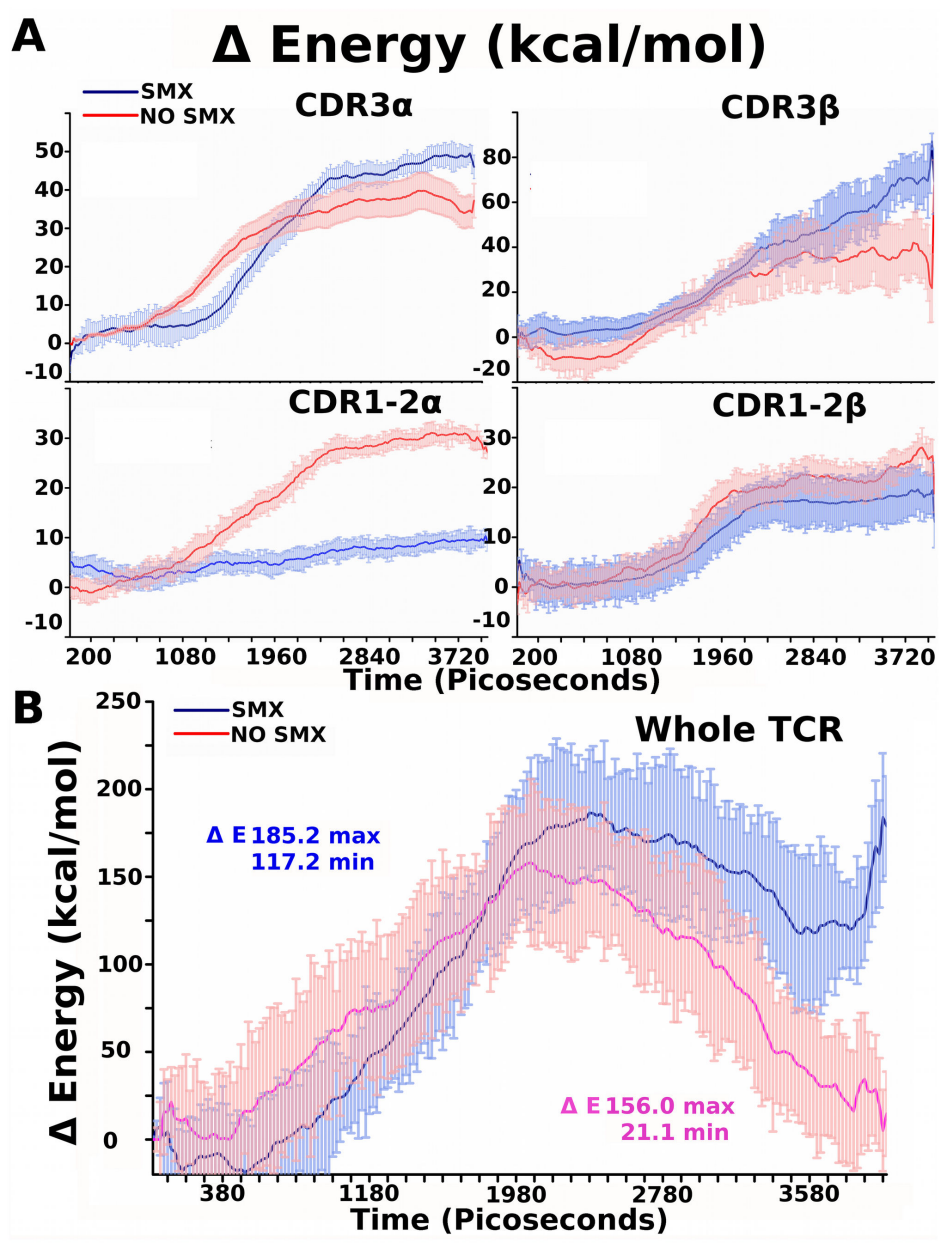
TCR and TCR Domain Energy Changes. **A**) Free energy change for specific TCR loops and pHLA interactions are given in kcal/mol. Graph title indicated TCR loop used from Figure 2A. Energies are Lenard-Jones and Coulomb short range between self only and pHLA, excluding solvent effects and neighboring TCR domains. These are summed for all trajectories and standard deviation represented by light colored bars, means as solid line. All energies, Y axis, are in kcal/mol. **B**) Free energy change for whole TCR, using only short range Lenard-Jones and Coulomb interactions for self atoms, excluding all other energies. These are summed for all trajectories and standard deviation represented by light colored bars, means as solid line. ΔE, total energy change for forward reaction taken at 3.8 ns, min, highest peak energy change as max. In all graphs, simulations with SMX, blue, without SMX, red. Short range cut offs in all were 9Å.

Energy changes were extracted from each trajectory and means calculated for domains making up the pHLA-TCR recognition interface or self-interactions between the TCR. Tabulated short range Coulomb and Leonard-Jones energies between specific variable domain and pHLA, self-interactions, but exclude solvation energies, which dominate the energy landscape are shown (Figure 4A). Changes were apparent from distant domains when SMX was present in the ligand-binding site for respective domains. Overall these showed, for summation of all shown variable domain energies, an approximate 10 kcal/mol higher affinity with SMX, however standard deviation overlaps significantly. For self-only interactions, the entire TCR was also analyzed for the same energies, excluding pHLA interactions as well (Figure 4B). These energies were truncated at interactions under 9Å in both summations, and show changes for the TCR, which correlate to functionality of domains. Contrasting this with total Gibbs energies, varied components are assigned to asses where particular energy contributions may arise, and to determine energy landscapes that may be missed in total energy analysis. This is highlighted for the TCR without SMX, which has a total energy change of only 21 kcal/mol, however a large 156 kcal/mol energy change between a free and bound conformation.

When only the CDR1-2α is analyzed, a higher positive energy is demonstrated which disappears when integrated over the entire TCR self-interactions. Standard error in this analysis is large compared to total energy, as alignment of separate trajectories is done by aligning individual energy summations extracted from single trajectories without zero point calculations. This analytical method therefore gives only rough perspective on trends, such as individual domains overall contribution to energy changes, without correlation coefficients determined to lower overall standard deviations. These rigorous calculations are done computationally using total energy and the pull force used to disengage the TCR in calculations with Gibbs free energy changes (Figure 3, Figure S1C). Additionally, this does not discern, for the CDR1-2α, if the energy difference observed is from pHLA contact or self interactions resulting from the binding process.

Both CDR3 show a moderate change, with α being more rigid as indicated by less fluctuation in the energy landscapes. A negative energetic contribution is induced in the CDR3β without SMX, however these fluctuate, also with SMX, indicting the loop has some degree of movement even in the bound state. This fluctuation is demonstrated through the standard deviation in Figure 4. The CDR2β shows little difference, only changing by 5-6 kcal/mol. Inclusive of standard deviations, the two energy curves overlap significantly. The liganded form only remains in a more rigid conformation than the free loop, weather bound or free in solvent across the trajectories.

From extracted energy, the greatest difference occurs in the CDR1 and 2α loop, with a 30 kcal/mol greater energy in the absence of SMX. Correlation with the large increase in total binding affinity does not correspond to any particular domain, but indicates a greater energetic change between TCR simulations in the CDRα and not the β domain. Comparison of the CDR3 loops shows a slightly higher affinity in both α and β for the SMX bound system, and no significant difference in the CDR1-2β loop. Summation of the two CDR3 loop changes overcompensate, or equal if standard deviations are accounted for, the difference observed in the CDR1-2α without SMX bound. It would be expected from simple interference in static hydrogen bonding induced in the TCR at the site of small molecule occupancy the site on the CDR2β should show the highest change. Energy means alone would indicate the CDR2β binding site as a ligand binding site rather than working by interference with the normal TCR pHLA hydrogen bonding. Also from a direct observation of the primary model (Figure 2 A,B), the small molecule does not come into contact with the pHLA in any direct way.

For the TCR as a whole, the energy change shows a large energy barrier for the unbound SMX state, approximately 156 kcal/mol. Using either end of the simulation as reference, this translates to roughly 132 kcal/mol in either the engagement or disengagement process. In a forward or reverse state, this would counter the favorability of either process. This energy barrier is present when SMX is bound but much smaller, suggesting larger conformational changes in the TCR without SMX or separate states in the overall pHLA recognition process. For the SMX bound conformation the energy barrier has a peak of 185 kcal/mol versus a final energy of 117 kcal/mol, giving a net change of 68 kcal/mol for the engagement of the pHLA. Disengagement however would incorporate the entire 185 kcal/mol making it much more energetically unfavorable than binding. This indicates SMX is able to affect an energy barrier present in TCR engagement of the pHLA, however does not give direct insight into the conformational change of the TCR. Summation of contributions between the entire variable domain energies, inclusive of the pHLA interactions, show these exceed the total energy changes. The difference is partially explained by the energy barrier in TCR only interactions observed, which includes a contribution from variable domain interactions with the constant domain. Additional long-range interactions, which have been shown in other studies to also play a role in protein-ligand interactions, are then also the remaining forces contributing to any energy differences between analytical methods observed [46]. Overall, these show that the majority of energy contributions to the difference in SMX induced affinity change are through changes within the TCR, rather than pHLA contacts.

### Root Mean Square Differences Between Models

**Figure 5:**
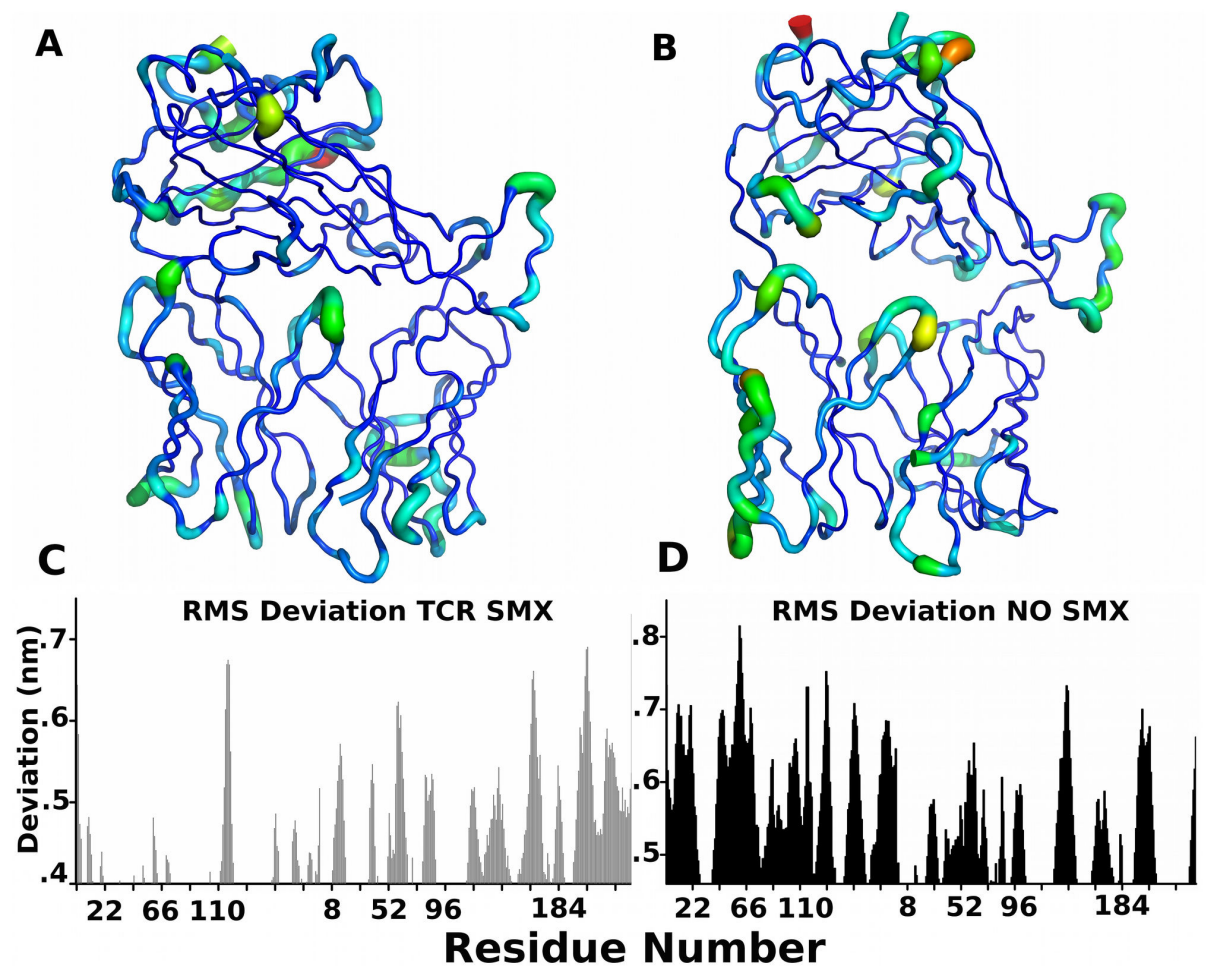
Root Mean Square Changes. **A**) SMX and **B**) no SMX RMSF displayed as B factors. **C**) **and** **D**) RMSD for respective trajectories in **A**, and **B**. Residues are numbered for the α then β domains, background is higher for no SMX, indicating less fixed side chains overall. Both truncate where STDV prevents differences from being observed. Linear distances are in nanometers in **C, D** for the Y axis.

As a cross correlation to energy differences, and to check other difference which may not have been apparent, both root mean square fluctuation (RMSF) and deviation (RMSD) across all trajectories were averaged. In both calculations a large background was apparent, indicating some flexibility in all parts of the TCR and differences associated with a random placement of amino acid side chains, which becomes amplified when averaging multiple trajectories [47]. An overall greater background was shown without SMX for the TCR indicating a less rigid molecule. Comparison of the RMSF, shown as a composite structural image (Figure 5A,B), correlated with the primary energetic observations. In the SMX bound structure, much less movement was observed in the CDR2α. Primary movements in this form were only at the distal ends of the CDR1 and 2α loops facing the pHLA and the constant domain of the TCR. Here, it was also observable that the inner CDR2α has more movement, suggesting it is part of the pHLA recognizing interface, which has been speculated from some x-ray structural analysis [48]. Additional loss of flexibility was also observed in the CDR3β which was not mirrored in the CDR3α loop.

Two other notable differences were observed in the constant domain of the TCR. One, a large movement in the constant domain helical region from residues α130-142 when SMX was bound. The second was with the constant region residues β220-230, which form a structure interacting with the βJ segment of the TCR. This structure places a core TRPβ225 in the center of a 10 amino acid loop, with a top distal ARGβ229 and is present in all TCR regardless of variable domains. Mechanically, this structure holds the ARG at a position, which intercalates between 2 adjacent residues in the J segment based on backbone hydrogen bonding, and forces the hydrophobic portion of the TRP in strongly rigid position against 2 residues of the J segment underneath the ARG site. This forces them inward and allows linear force to be applied to the J segment, itself, showing a role in maintaining the overall force along the segment spanning from the CDR3β to the constant domain in all TCR.

Comparing RMSD show an expected degree of correlation with the RMSF (Figure 5C,D), and are used as a cross control [49]. Differences are a greater degree of distance movements in the α domain without SMX. In the presence of SMX, there is almost a complete loss of movement in all three CD1-3α from residues 1-100, but also much less movement with β loops. Large changes begin at the distal end of the βJ segment at residue 115, showing a greater movement in the constant domain than in the absence of SMX. Additionally there is an observable shift in residues close to the CDR3β loop, from the distal end residues 94-98 to residues 85-90 and higher on the J segment, when SMX is bound. Movement is also detected in RMSD for the α domain, from residues 111 to 121, which represent the end of the αJ segment, while the remainder of movements are small but sharp along loop ends, or from the constant domain above the top ends of the α/β loops in the SMX bound TCR. This indicates a much more rigid TCR when SMX is bound, along with a switch between primary recognition of the peptide from the CDR3β to the CDR3α, and a change in the constant domain in both the subunits.

### Linear Movements Across Trajectories

**Figure 6:**
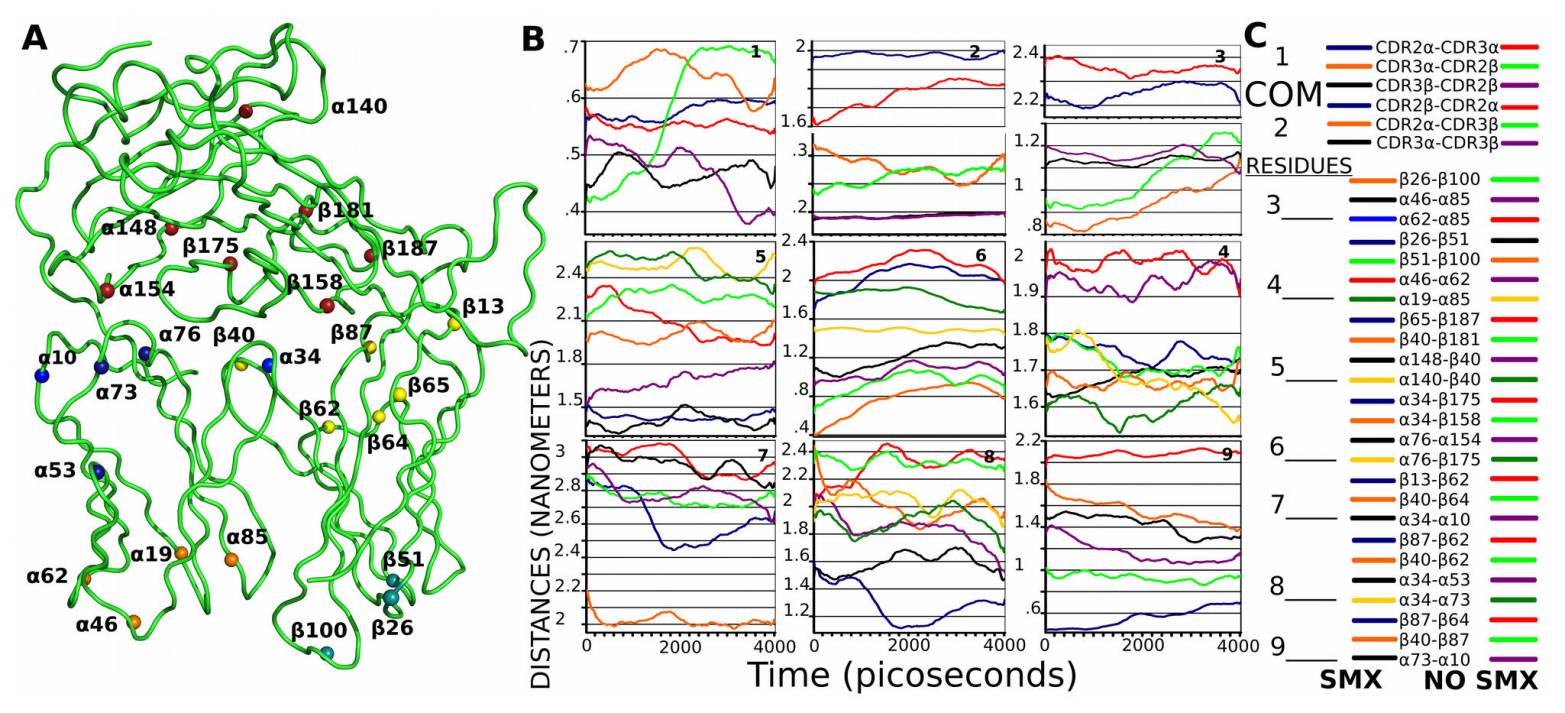
Distance Changes Between TCR Domains and Residues. Positional changes between models. **A**) Individual residues used in distance determinations shown across the entire trajectories. Each colored ball represents the labeled residue used **B**) mean averaged distance change for all trajectories for COM defined in text (Figure S2) as CDR2α/β, CDR3α/β, or residues shown in **A**). **C**) Residue pairs are indicated representing distances shown between two points in model, line colors correspond to graphs in **B**), numbers at left correspond to graph numbers at upper right corner of each graph in **B**. SMX lines on left, no SMX, lines on right. COM are graphs 1 and 2,each with 3 line comparisons. All single residues the remaining graphs. Line to number in **C**, last compared residue on corresponding numbered graph in **B**.

We next analyzed direct movements between atom pairs and COM for specific domains (Figure 6, Figure S2). Specific positional differences for charged atom pairs could be compared, with standard error ranging from 1 Å to 0.05 Å (Table S1), based on overall movements of the loops indicated along with the conformational flexibility of individual amino acids used for the determinations. For the variable domains, COM was determined by breaking regions into amino acids sets representing various loops. Residue to COM definitions were respectively, CDR2α residues 40 to 68, CDR3α residues 84 to 91, CDR2β residues 46 to 74, CDR3β residues 95 to 105. COMs for the 2 CDR3 loops were only 2-3 Å apart, however these COM coordinates did not change when more amino acids were included as the loops are relatively linear, running strait up and down from the constant domain direction to the pHLA interface on one side, and slightly offset moving towards the respective J segment.

Comparisons of these movements for COM distances for both trajectory sets from a pHLA bound to free showed the two CDR3 loops did not move respective to each other. The CDR3α and CDR2α only show a small 1Å difference, indicating minor change when SMX is present, from a pHLA to free state. This same movement is only half as large comparing the CDR3β to CDR2α distances, only 0.5Å, with error estimates of 0.15Å. The largest change was from CDR2β to CDR3α COM, a change of 3Å, which also indicates the CDR2β moves at an angle rather than contracting as the CDR2α does. Correlating with differences observed in RMSF or RMSD show a change in the whole CDR2α when SMX is absent. This can also be observed in the CDR2β and CDR3β COMs, which show a change less than 1Å from the pHLA bound to free TCR state, and the two extremities of CDR2 COM movements respective of each other, of only 2Å.

When SMX is bound, this inter CDR2 movement is absent, showing the CDR2β is more open. Overall COM movements show a more rigid structure, held in a conformation that is 1-2 Å more open, respective of the variable β domain. The variable α domain retains a small degree of movement with ligand present, however itself is also much more rigid. With the CDR3α domain, as shown in graph 1 of Figure 6, between the CDR2β, the largest observed movement is tied in with the disengagement of the TCR when SMX is absent. Correlated with the RMSD or RMSF, this attributes a portion of Figure 5B and D to the disengagement process.

Analysis of movements between charged pairs were grouped into upper and lower loops of the variable domains, and constant domain atoms above loop ends at either variable region. This latter was used to measure distances from either the α or β variable domains in respect to the constant domain, or respective variable domain loops and each individual grouping is shown (Figure 6A). Distances are calculated as 6 sets, allowing the movement of loop ends to be shown with respect to each other at bottom α/β, top α/β, and top α or β to constant domain, represented as the mean across all trajectories. Graphed distances (Figure 6B) are grouped together based on scale or upward and downward movements.

The most notable effect from the presence of SMX was an apparent inhibition of a fold of the top of the CDR2β downward, which was fixed even after the TCR dissociated from the pHLA, placing LYS63, ASP64 and LYS65 several Å further towards the pHLA-TCR interface. These three residues have the highest standard deviation, 1Å, as the terminal N or O were used for distance measurements. This shows difference in conformation, where LYS63 is moved pointing downward while LYS65 only moves slightly adjacent in orientation.

Variable domain to constant domain movements, show a switch between bound and unbound SMX. In the presence of SMX, the tops of α loops move close to the constant domain 5-6Å, while in the absence the β loop tops move towards the constant domain 3-4Å. This is a switch mechanism, however, comparison of the top distal ends of all 3 loops from either constant domain shows the movements are not entirely grouped together. The loop spanning CDR1 to CDR2 moves not only upward, α34 and β40 with or without SMX respectively, but also towards the other respective variable loops. This shows some sort of compacting of these loops against the other respective domain variable loops. These movements also carry some rotational aspect, as comparisons in either case to the primary CDR3, J or CDR1 spanning loops differ for residues α76 and α10 or β87 and β13, showing an offset in direction along the outer variable domains. All movements between loops either at the top or at bottom are not uniform in respect to the same variable region, yet correlate to movements from equivalent top to bottom.

Small differences in the two systems, exemplified from either α19 and β51 or β26 of 2Å with or without SMX are translated to much larger differences at the tops for the CDR2 or CDR1-2α/β spanning loops of around 5-8Å. This shows the CDR1 to 2 movement is tied together, induced by engagement of the pHLA. Minor differences respective to CDR3 loops, show the β moving 2Å on pHLA recognition only with SMX, and the α fixed this distance. This CDR3 movement is reversed without SMX, and much larger movement is observed for the CDR3α than the CDR3β equivalent with SMX. A small degree of random fluctuation is also observed with the entire set of loops, restricted to the distal ends of both the tops and bottoms as shown by oscillatory movements of around 1-2Å. Together these suggest a degree of randomized flexibility regardless of ligand occupancy, but a much more rigid state of the TCRVα with SMX.

### Principal Component Analysis

**Figure 7:**
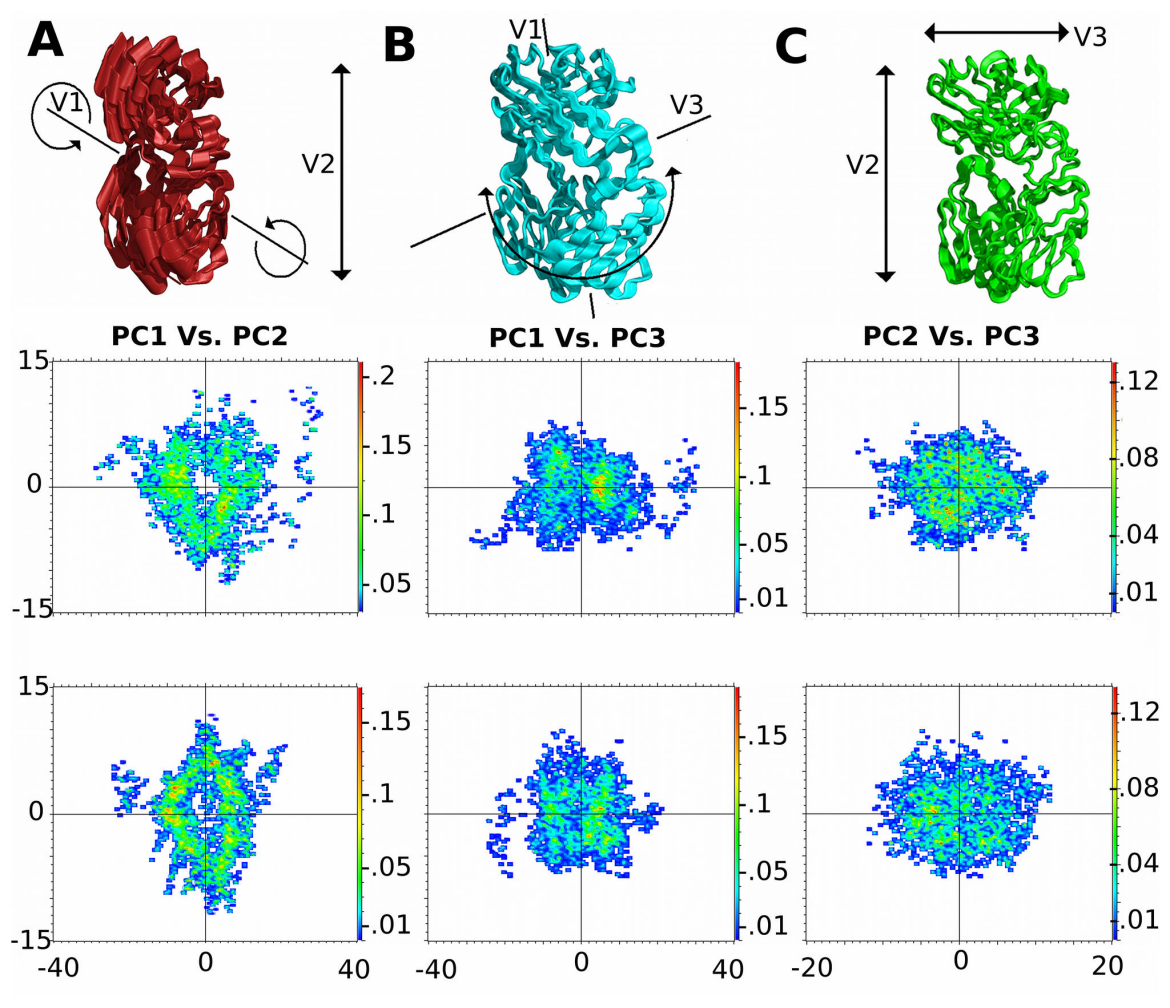
Principal Component Analysis of TCR Disengagement Process. PCA analysis of entire TCR. **A**-**C**) Principal motions as defined by PCA and corresponding energy plots projected onto these for the first 3 primary vectors defined in top of graph as Y, then X dimension indicated, scale is in nanometers. Top plots, with SMX, bottom plots without SMX. Heat maps are in kcal/mol. Pinpointed domains or residues are shown in Figure S5. Highest energy peak for PC1 vs. PC2 is αJ loop with SMX, both loops distribute this energy equally without SMX. Primary motion, vector 1, is a twist dominated by the constant domain in SMX and NO SMX analysis.

**Figure 8:**
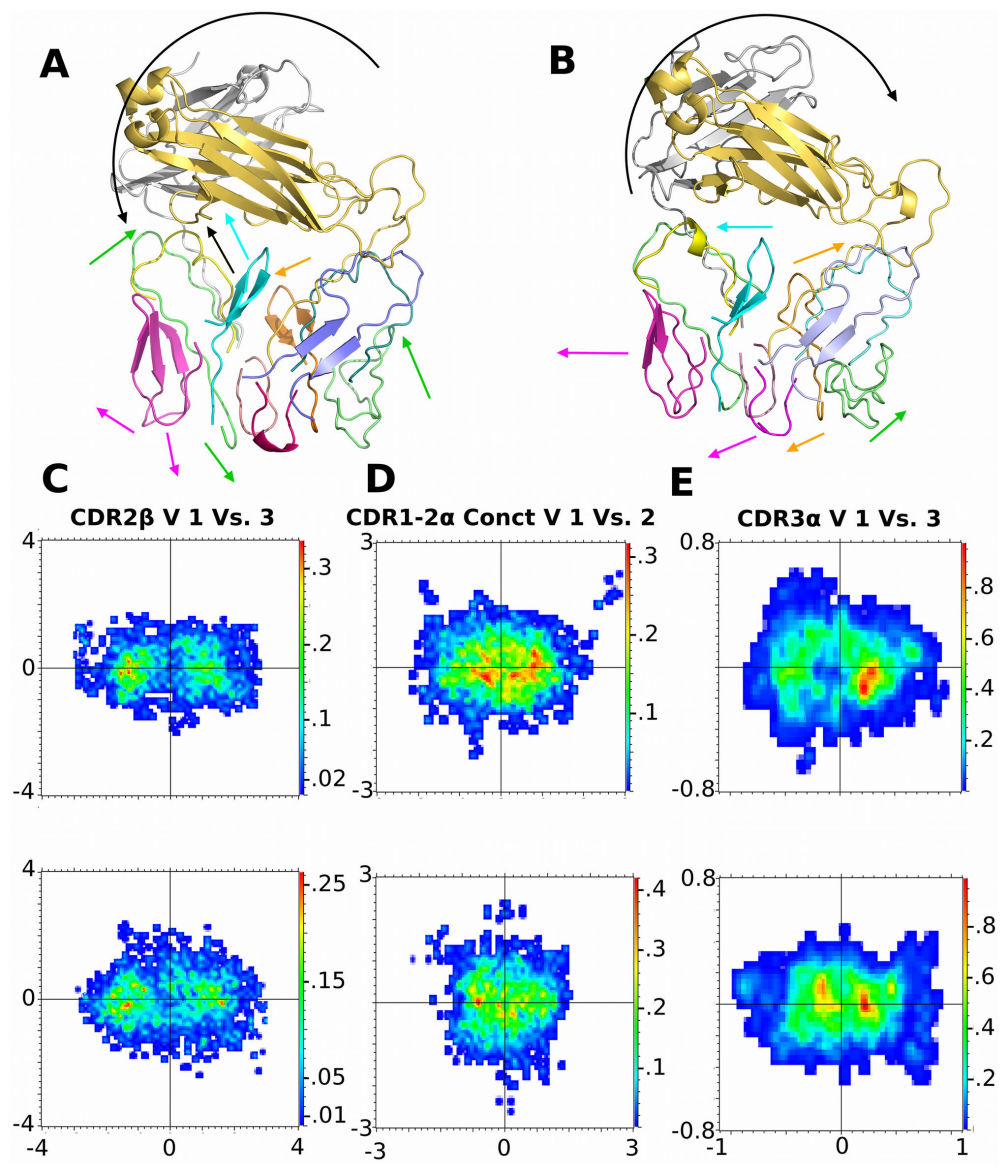
TCR Domain Motions from PCA. **A**,**B**) Summary of principal motions for SMX and no SMX trajectories from PCA of individual domains respectively. Colored arrows correspond to same colored loop, except black arrow in A, representing yellow loop between CDR2α and 3α. Arrows are drawn against an X-Y-Z dimension, arrow heads point in direction of movement. **C**) CDR2β, lower pink in **A,B.D**) CDR1-2α upper connecting loop, turquoise in **A,B.E**) CDR3α loop, salmon in models. Highlighted PCA for individual domains in title of plot, V, vector used in Y and X axis, dimensions are in nanometers. Heat maps are in kcal/mol. SMX, top plot, no SMX lower. These are used to illustrate the clearest differences. All individual domain PCA are shown in Figure S6. X and Y dimensions are the same for each plot.

We conducted principal component analysis (PCA) across the first three primary vectors, with total energy projections onto each of these averaged, for each trajectory set [43,50,51]. Means for all trajectories were calculated, and vector directions as linear or rotational determined visually using filtered trajectories for each principal component (PC) (Figure 7,8, Movie S3-5). For individual linearized vectors, energy break downs were 54.0 and 47.4%, 18.0 and 19.0% and 18.0 and 23.7% for PC1 to 3 respectively with and without SMX. A switch in Z and X-Y plain movements, based on energy is observed with PC2 and PC3, however vector numbering remains the same to allow for direct comparisons between PCA maps. Individual atoms and domains were pinpointed and individual domains corresponding to the same residues for COM calculations, with the additional grouping of residues α23 to 39 and β30 to 45 making up the upper inner loop between CDR1 and 2 for each domain. This later grouping is based on large movements not expected, but observed in linear and RMSD or RMSF analysis.

Highest energy points from plots of PC1 and PC2, which represent a rotational and stretching in the z direction, are for the αJ segment in the presence of SMX. These shift to a more even distribution between J segments incorporating the CDR3, CDR2 loops, and a bias towards the CDR1-2β spanning loop in the absence of SMX.

Projections along the PC3 and PC1 components however show a much wider difference between SMX bound isoforms, where the bias is more towards the CDR2 and 3α, with a minor high energy peak along the CDR2β corresponding to contact residues [43,51]. Along the PC2 and PC3 plots, energies correspond to individual residues contacting the pHLA and residues, which form hydrogen bonds, or hydrophobic interactions with the constant domain, directly from the α/β domains of the TCR in the presence of SMX (Figure S3). With SMX, a peak bias along this last projection is towards the β CDR1-2 spanning loop, and a much larger overall contribution from α constant domain residues. This further illustrates a switch mechanism from β to α TCR domain movement.

A PCA for each domain retained the same directional bias correlated with energy level for both the α/β CDR1-2 connecting loops and CDR2β only, showing these contribute significantly to the total energy distribution and motion associated with pHLA recognition. This additionally shows the CDR2β as moving primarily in a rotational, rather than through linear motion along with the other two upper loops. The upper CDR1-2 spanning loops retain overall motions, and thus energy contributions with or without SMX towards the corresponding variable domains. These two loops switch from upward and towards the other CDR tops to only towards the other loop tops, β with SMX and α without respectively.

All other domains showed vectors moving along different rotational or translational axis relative to their intrinsic motions. All movements discerned from PCA and linearized analysis for respective domains are illustrated as a composite image (Figure 8A,B) in the presence and absence of SMX. Main differences are complete switch in direction of the rotation of the constant domain around a central axis through the TCR towards the α domain when SMX is present, from a rotation towards the β domain in the absence of a ligand. With α loops a much larger movement upwards towards the constant domain with SMX, when engaging the pHLA, is seen than with the β without SMX.

A summary of these motions and associated energies (Figure 8C,D,E, Figure S4), show each loop moving almost independent of each other across trajectories, differing only in domains based on the presence of SMX. One notable movement, and corresponding energy difference, is highlighted by the upper loop between the variable CDR2 to 3α. In the presence of SMX, this loop moves inwards against adjacent α domain loops and the constant domain, while lacking almost completely any change without SMX. The lower portion of this loop moves inward after disengagement of the pHLA when SMX is bound, with only a more random fluctuating pattern across the trajectory without SMX. A filtered set of trajectories (Movie S6, S7) for the TCR as a whole highlight all these movements. A final determination, resulting from PCA analysis was an observation that the entropy of the two different TCR conformers remained the same, approximately 5.6 kcal/mol, allowing for a complete energy distribution to be calculated (table 1) [52].

**Table 1:** Total free Energy, entropy and enthalpy calculated for the TCR across all trajectories.

**Energy Distribution**	**ΔG**	**ΔH**	**-ΔS**
SMX	139.77 +/- 15	134.15 +/- 15	-5.620 +/- 0.028
No SMX	23.79 +/- 5	18.122 +/- 5	-5.669 +/- 0.020

### Pearson Correlation of Energy and Linear Movements

From PCA alone, large rotational energies dominated the overall movements of the TCR, and motions related to the constant and variable domains have not been described previously. To further correlate motions determined for the TCR and sub-domains, respective of constant domain positions, Pearson’s r-value was used to check dependence of varied distances (Figure 6, Table S2) against the first 3 PC vectors, listed for bound SMX and the un-liganded TCR respectively [53,54]. Additionally, we also determined these for the CDR2β, as encompassing site of SMX induced switch in TCR affinity. For the upper CDR1-2β, residues β40-β181 represent an upward and twist motion with a change in r-values of 0.217 to 0.295 for PC1, -0.232 to -0.202 for PC2 and -0.421 to 0.206 for PC3, thus main X-Y plain change dominates the energy changes between the SMX to non-SMX state. For the equivalent α loop, residues α34-β175, these correlations are higher for rotational energy, but indicate a change in the absence of SMX to primarily the X-Y plain. Equivalent r-values were 0.790 to 0.795 for PC1, while stretching PC2 changed to 0.360 from 0.476, and -0.283 from 0.451 for PC3. Together indicating a switch from X-Y, up and constant domain tilt, to a rotational only movement in the presence of SMX.

Effects on the ligand binding domain, residues β65-β181, were loss of rotation with SMX and switch to X-Y movement, with r-values of 0.387 and 0.056 for PC3, while PC1 and PC2 showed correlations which switched to -0.365 from -0.871 and -0.719 from -0.795 respectively. This showed an expected correlation with respect to TCR affinity from the other analyzed data, where rotation occurs from the variable β without SMX, but also the X-Y plain change is significant. Analysis of all positions shows inhomogeneous correlations to energy, which mostly translate to relations between varied loops distal ends, switching from β to α loops with SMX. This contrasts a domain only analysis, showing small regions, or individual amino acids, correlated more with X-Y plain and Z direction, which average out between the variable domains for bound and unbound TCR forms (Table S2).

### TCR Hydrogen Bonding Changes on pHLA Recognition

**Figure 9:**
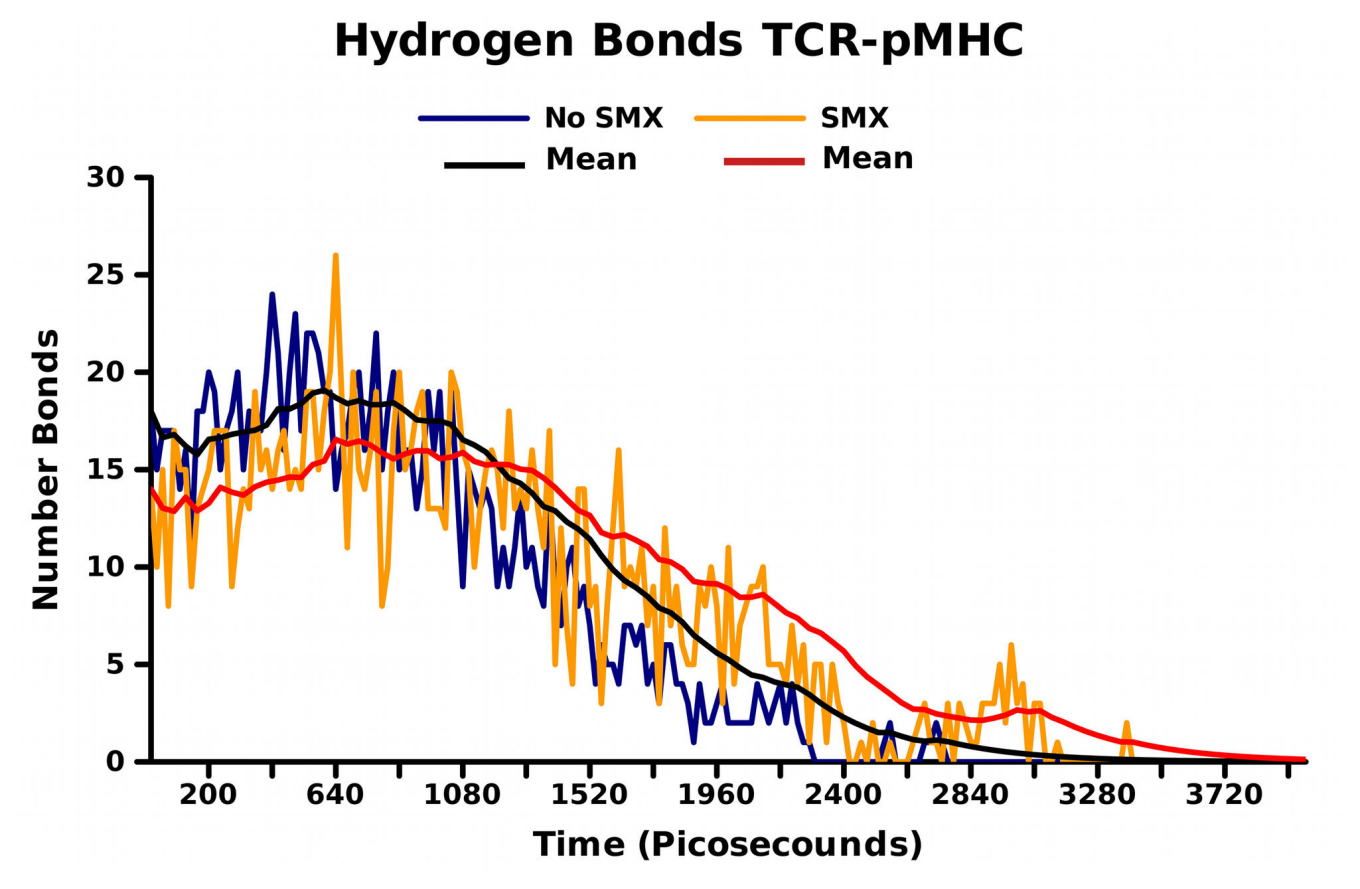
Hydrogen Bonds. Number of hydrogen bonds across entire trajectory, set at 3.5Å distance maximum, between the TCR and pHLA. Trajectory 5, and Trajectory n5, for SMX (orange) and no SMX (blue) are graphed. Means are shown as solid lines, red SMX, black, no SMX. Hydrogen bonds are release more rapidly without SMX, and are the same in all trajectories, with differences in time frame at 20 picosecond intervals only over the first 2 nanoseconds for either trajectory set. This reflects bonds that change randomly at the pHLA interface, even before the TCR disengages. All bonds found through the first 300 picoseconds are listed in table S3.

Direct hydrogen bonding in analysis of protein energy changes is often used as the most common proof or analytical tool to show changes in static structures, such as x-ray, to demonstrate differences between varied states [49]. To analyze these same interactions comparative of the many available pHLA-TCR structures, we used the first 300 frames for each trajectory to determine fixed and oscillatory hydrogen bonding with the pHLA, and internal TCR hydrogen bonding thought to be relevant (Figure 9, Table S3). Respective of the pHLA, a change from a total of 25 to 21 without and with bound SMX occurs for the HLA, and 8 total in both for direct peptide recognition. However, at any one time frame, only 12 with SMX and 15 without for the TCR-pHLA, with 4 to 5 for peptide alone, hydrogen bonds are present. This translates to a decrease of 2-3 hydrogen bonds when SMX is bound directly between the TCR and pHLA averaged.

Hydrogen bonds between the TCR and peptide oscillate between the indicated frames much less with SMX. Main differences are with peptide ARG5 becoming fixed against the CDR3β, and a change from backbone O or N recognition, to direct amino acid recognition in the CDR1α with SERα21 and ASNα23 to peptide ALA2 or THR3. The CDR1 and 3α loop forms hydrogen bonds from ASNα87 and GLNα88 with HLA GLUα53, GLNα55 and GLYα56, peptide ALA2, THR3 and terminal NH3, that change to a recognition of these pHLA residues from the CDR3α only when SMX is absent. This is primarily through changes in CDR1α ASN23, SER21 and THR20 moving more over the peptide. Larger changes seen with the CDR2α and the HLA itself, are from TCR ARGα43 to HLA GLNβ60, ASPβ62, LEUβ63 with, and TCR SERα44 and ASNα45 to HLA GLNβ66 and ASPβ62 when SMX is absent. Overall, recognition from the α domain for the entire pHLA switches from CDR2 and 3 to CDR1 and stronger CDR3, when SMX is bound.

From the CDR3β, a shift from slight recognition of the HLAβ to α helix is apparent. The CDR2β retains the same amount of hydrogen bonding with the HLA, 4 total, only changing overall residues between the two proteins, which make up these interactions. In the bound form, SER53 hydrogen bonds with HLA LYS65α, switching from TCR LYS54 recognizing HLA residues GLNα55 or ASNα60 without SMX. In both, induced bonds oscillate, and are present only in 50% or less of bound frames in trajectories. In the absence of SMX, there is a slight shift of SER53 to ASN69, moving it away from the HLA, but also effects from the overall placement of the CDR2β over the HLA.

Comparing TCR internal hydrogen bonds from self α/β loops shows an increase of 5 and decrease of 5 for the α/β, respectively with SMX. Our data shows that direct hydrogen bonding with the pHLA from the TCR is not highly correlated with the overall binding affinity, or energy changes observed from the engagement process. More importantly, the mode of binding seems to be tied in with TCR-pHLA affinity, and the rigidity of the hydrogen bonds involved in a solvated system, highlighted by the hydrogen bonds in peptide recognition. With SMX present, the peptide amino acids recognized become rigid, but also bonds from the variable α loops and the HLA itself are important and more rigid. This is observed by duration of the hydrogen bonds across the trajectory as the pHLA is disengaged in Figure 9.

We visually inspected models to determine the network of residue interactions affected by SMX binding, to determine how these changes are transmitted (Figure S6, S7, Movie S8, S9). In the absence of SMX, respective of the β domain, a small network of hydrogen bonds is formed between primarily LYS65 and both SER82 and TYR91 or ARG36. Through ARG36, SER88 and PRO39 bonding the inner CDR1-2 loop is pulled close to the other β loops. This also allows for further hydrogen bonding, between loop distal top ends, bonding with the constant domain. A net result additionally holds the constant domain fixed to the J segment from the functional domain comprised of residues β220-230. These are all mediated through adjacent loops affecting each other through cross loop hydrogen bonding.

Bound SMX interacts directly with the backbone of LYS65, and THR56, partially the side chain of ASP64, and with the side chain of SER53, LYS63, LEU 67, ASN69 and TYR57. For the latter there is a dependency of TYR57 (Figure 2B) in a position found only in the unbound, respective of SMX, form of the TCR, which switches to a stabilized bond with SMX and the inner loop backbone atoms of the CDR2β. The residue ASP64 oscillates between a salt bridge with SMX sulfur, solvent or adjacent backbone hydrogens. The main switch occurs through this ASP residue, and LYS65 bending the loop forward. Hydrogen bonding from LYS65 to SERβ82 and ASPβ87 which disrupts the network with the inner CDR1-2 spanning loop, causing the primary recognition and tilting of the overall constant domain to occur. Additional effects from a re-arranged hydrogen bonding network originating from this point also cause the β220-230 to release the J segment.

### Ramachandran Plots

**Figure 10:**
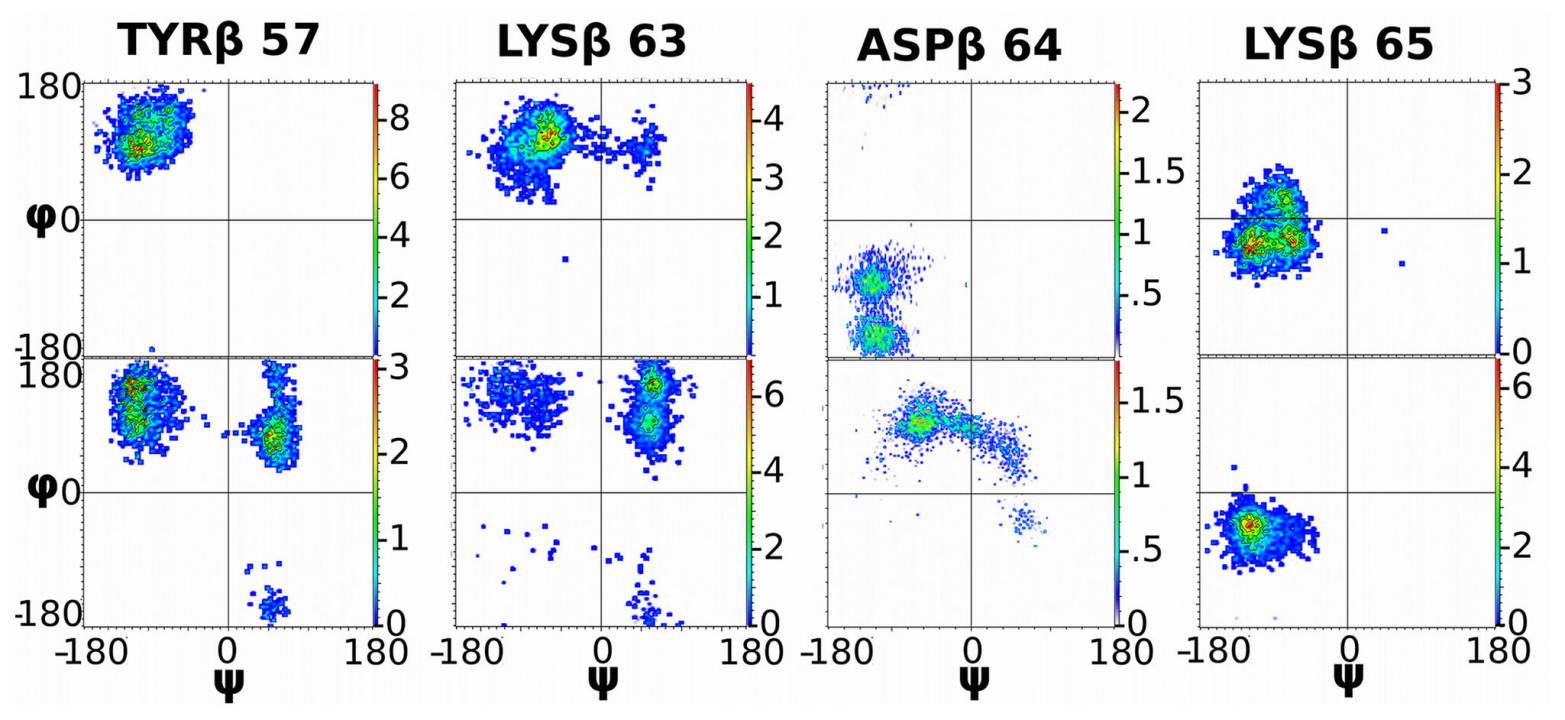
Ramachandran Plots. Ramachandran plot of 4 primary residues on CDR2β affected by SMX binding in loop. Plots are for entire trajectory, and show also differences in bound TCR to free TCR. Φ and ψ angles are labeled, axis are from -180 to 180 degrees. Top are with SMX, bottom without. Intensity, right color scale, indicates scaled intensity factor relating percent occupancy across all trajectories, red, highest occupancy scaled to number indicated. All data in each plot represent 100% of angle occupancy summed from all trajectories in set.

Ramachandran plots for the residues making up the primary switch (Figure 10, Figure S5) highlight the differences of these residues in the SMX and non-liganded form of the TCR, allowing another measure than those already employed to illustrate these overall changes. Plots were generated for residues around the SMX site thought to be involved. In all, most of the same residues found in hydrogen bonding with SMX also show shifts in angles around the amino acid side chains.

Hydrogen bonding, or large linear differences in position of an amino acid can sometimes not change the angles found, which is reflected only in 1 residue plotted, ARGβ36. This residue is 3Å displaced between structures with and without SMX, however this does not show in Ramachandran analysis. For TYRβ57, the structure is fixed with SMX in a position only observed in the unbound TCR without SMX. From direct analysis this is not apparent, only after observing visual trajectories can this be seen. For LYS65β this is also an effect even though angles are slightly altered, while LYSβ63 and ASPβ64 show a significant change between conformations. For LYS65β, the observed difference is reflective of randomized changing bonds, while SMX is present primarily between SERβ82 and ASP87, while this residue is hydrogen bonded between SERβ82 and TYRβ91 or ARGβ36 without SMX. This randomized movement is not significantly reflected in the overall plot, however small changes are observable between the two conformations.

## Discussion

Work here focused on a TCR isolated from a pool of TC showing a proliferative response to SMX. These were originally isolated from whole blood mononeucleocytes from a patient showing skin eruptions when administered SMX. The aim of this work focused on non-covalent interactions with the TCR, as related to ADR, and the TCR was selected based on having no clear direct interaction of the small molecule with the pHLA interface [8,12,55]. Our goal was not to define the actual culprit for the particular patient, which may include several other factors giving rise to ADR. Rather we wished to utilize the highly reactive small molecule SMX as a probe to characterize specific interactions with TCR not completely explained from other studies. This particular TCR type, and identified interaction should prove useful to ADR research, and the immunological community as a whole, providing direct characterization of a small molecule interaction that can elicit a change in the TCR-pHLA mechanics. While this may not be the cause of the reaction in the original patient, the particular Vβ20-1 has been associated with a range of autoimmune diseases, also having unique features such as being the only TCR to bind TSST-1.

As ADR themselves mimic autoimmune disease to a high degree, it makes this particular TCR a valid target for characterization related to either type of medical problem [9,26,27]. Like autoimmune disease, different ADR may also have a more complicated nature for onset. This is highlighted through comparisons of such diseases as Sjögren’s syndrome and papular skin eruptions, and drug induced eruptions on lips of patients that has been shown to occur [17,23-25]. Our model determined Lamininα2 as a highly affine ligand for the TCR used, a protein often associated as over-expressed in salivary glands of Sjögren’s patients. Nevertheless, like this autoimmune disorder, several other factors such as tissue damage through other means, and several other effector immune cells make direct cause and effect analysis of these models difficult. It may be these TC containing this TCR are only part of the overall causative mechanism behind the pathology. However ADR has been shown to mimic inflammatory response at several levels including cytokine profiles produced by associated effector cells involved, with TC isolates present in all cases. A further example of complexity, in the case of Sjögren’s syndrome the onset was shown to be caused through initial Ig production against a Rho-GTPase, initial tissue damage, and accumulation of nucleotides. The TC containing Vβ20-1 are secondary, however are always found as salivary infiltrates and have been shown to sustain the inflammatory response [6,16,24]. In this entire scheme, even further complexity is observed with patients often showing an overproduction of Lamininα2, a basement membrane protein expressed at nerve tissue, some basement membrane, and salivary gland sites.

Further hindrance in defining overall molecular aspects of ADR arise from lack of a direct understanding of TCR mechanics in the entire process. Currently conflicting results exist on what constitutes differences between signaling and non-signaling TCR based on structural studies [4,32,36,39,56]. Related to ADR or autoimmune disease, these have focused on the pHLA interactions themselves, and largely neglected TCR conformations, primarily as HLA types vary between individuals, while TCR subtypes do not [1,10,14]. These differences are highlighted in reviews analyzing dozens of pHLA bound TCR which show TCR orientation over the pHLA that differ drastically in placement of all CDR loops. There are also no structures available that indicate any interactions outside of the pHLA would cause effects on the TCR mode of antigen recognition. A secondary result of our work here compares two pHLA recognition events, where the peptide and HLA do not vary, only the TCR conformation. This allows a unique view of changes that occur in the pHLA recognition process, which can be directly related to the ample structures available. These changes are described in detail, related to this particular TCR, and are based on SMX induced changes. While this constitutes a perturbation in the normal system different from the processes as they are usually described it may be a restricted case. Regardless, this can also provide a framework for already available data to describe and alleviate discrepancies from multiple conflicting results in immunology related to this process.

Our data shows for the particular TCR analyzed two completely different modes of pHLA recognition. This encompasses primary recognition and alignment of the whole TCR via intrinsic constant CDR1αβ and CDR2αβ domains over the HLA. In a normal mode, once recognized, the Vβ20-1 domain then relaxes, and the CDR1β and 2β move 1 to 1.5Å away from the variable domain COM, primarily the CDR3α/β loops mid region. By doing this, a few amino acid side chains in the CDR3β terminal portion are given wider degrees of movement, which allow recognition of the presented peptide. This small domain movement also allows the CDR1β and 2β to recognize the HLA better, forming hydrogen bonds with residues on the HLA α helices. Upper portions of the CDR1 and CDR2β form direct hydrogen bonds with residues starting from LYSβ65, 63 and ASPβ64 that signal along a network across the upper edges of the Vβ domain of the TCR. These culminate in hydrogen bonds with the constant domain, also mediated by bringing the inner CDR1-2 spanning loop closer to the other Vβ loops. This tilts the constant domain to the Vβ domain.

With SMX bound in the CDR2β, hydrogen bonding translated to upper portions of the α domain changes equivalent hydrogen bonding on one side of the TCR, moving similar amino acids above the CD1α and CD2α loops 5Å closer, effectively tilting the entire TCR constant portion to one side along an axis between the two variable domains, now toward the Vα. This is accomplished also by releasing the domain comprised of residues β220-230 from the βJ segment, which is held in place normally. The two J segment differences are reflected in the PCA analysis, which shows a switch to the αJ segment as the highest energy peak in PC1 vs. PC2 energy plots.

On TCR comprised of Vβ20-1, a pocket exists in the CDR2β, which has a strong affinity for SMX. This pocket has only in prior studies been shown to bind TSST-1, acting as a superantigen by TCR-HLA cross-linking outside the peptide groove [40]. The highlighted variable α/β switch in recognition and tilting of constant domain is controlled through this pocket. In the presence of bound SMX, 2 amino acids at the top of CDR2β, LYSβ65 and ASPβ64, form alternate hydrogen bonds with the inner CDR1-2β loop. Additionally, TYRβ57 is held away from adjacent CDRβ loops. Residues in all these loops then also switch to higher residues in the adjacent CDR3β and J segment, or constant domain. A rather large CDR2β terminal portion is also prevented from holding CDR3α with SMX bound, allowing the loop to move closer against other CDRα loops before pHLA recognition. In this SMX bound conformation, the TCR Vα domain then forms hydrogen bonding with the TCR invariant constant domain, and the CDR1α and 2α loops dilate from the CDR3α as with the β loops of un-liganded TCR recognition. These dilations however are only in the distal ends of these loops, while the central portions remain closer together. In a similar fashion, the CDR3α terminal residues then recognize the peptide more specifically than in the normal recognition mode, and an overall switch from CDR2α and 3α, to CDR1α and 3α recognition occurs. Net effects are a 7 fold increase in binding affinity for the same peptide and HLA in the presence of ligand.

Implications for the mechanics of TCR recognition from this data show the hydrogen bonding with the pHLA is not directly determinant of the affinity [31,56,57]. It is demonstrated the mode of binding by a TCR is more important for the affinity, as the hydrogen bonding with SMX has a net loss of 3 hydrogen bonds however has a much higher affinity for the same pHLA. The change of tilt for the constant portion of the TCR, in respect to the variable domains, indicates a possible “on” or “off” mechanism for TCR signaling. Published x-ray structures for whole TCR-CD4-pHLA complex show docking of the Ca2++ inducing signaling CD3εγδ portion of the entire macromolecular signaling complex to the β domain only [58,59]. This would indicate TCR signaling is not directly affinity based, rather conformational based and the way the Vα or Vβ primary recognition of the pHLA occurs would dictate signaling through tilting of the constant domain. Our case however may be specific for the TCR used in the MD experiments, and without definitive proof of the properly bound CD3εγδζζ/TCR complex, this is still speculative. As this TCR was isolated from TC proliferating when SMX is present, but failing to proliferate when not with the same antigen presenting cells this remains likely however. This is furthered, as most isolated T cells do not respond to SMX in proliferation assays [11,55].

We also show that the affinity change is not dominated in this case by global loss of movements induced in the TCR, by comparing entropy and total affinity changes across the trajectories. Entropy remains the same in both TCR conformations in our MD simulations (Table 1), showing solute effects are roughly equal, thus enthalpy dominates the affinity change observed [32,60,61]. In individual domains and whole TCR energy summations, the difference is explained by the non-SMX bound TCR having an energy barrier equal to the positive change in energy, thus canceling any effects through conformational changes on pHLA recognition. These motions are exemplified for both TCR forms by large rapid change on pHLA disengagement (Figure 6), such as between the CDR3α to CDR2β loop, larger constant domain tilt with SMX (Table S2, Movies S6-S9), and large but continuous random movements in the α loops overall without SMX (Figure 5). Comparatively the changes in motion between TCR with and without SMX are only different domains, loops or J segments but remain equal in respect to motion alone. For the SMX bound TCR, these energies do not include a large negative energy barrier, and thus the recognition process retains a large positive contribution from the TCR conformational changes.

In conclusion, for our specific TCR comprised of Vβ20-1 and Vα17-1, a clear switch in mode of binding is demonstrated when a proper ligand, here SMX, occupies a large CDR2β pocket. This may imply a natural ligand exists, however can also simply be an artifact of interaction with SMX. We also show affinity change is not straight forward and based on differences in pHLA hydrogen bonding alone, rather it includes a complex system of hydrogen bonding internal to the conformation of the TCR as it is held in a pHLA bound state. This is shown as the majority of energy associated with TCR-pHLA interaction is dominated by energy internal to the TCR and not pHLA interactions (Figure 4). Pearson’s correlations of energy and movements show the larger domain tilt as dominant, with varied contributions from loop ends themselves only switching between variable domains in the presence of SMX. This would indicate a signal to cause domain tilting, and the tilt of the domain to the α or β variable side is the dominant feature in this TCR related to affinity (Figures 7, 8). Overall, our data shows this particular TCR can undergo large conformation shifts, suggesting competing hypothesis need to be validated with other models. One, specific differences between TCR subtypes not related to variable CDR3 loops play roles in pHLA recognition, or second that generally TCR themselves do utilize larger conformational changes in their overall pHLA recognition process distinguishing a signaling or non-signaling conformation. Our model described here fits this second hypothesis accurately, but cannot exclude unique features of this TCR without additional simulations utilizing completely different TCR subtypes. For this particular TCR, SMX is clearly shown to cause a change in TCR conformation and pHLA affinity, thus indicating TCR containing Vβ20-1 have a roll in drug allergy.

## Materials and Methods

### Initial Model Generation

Models were generated using Swiss-modeller [62] from the sequenced TCR α, QQGEEDPQALSIQEGENATMNCSYKTSINNLQWYRQNSGRGLVHLILIRSNEREKHSGRLRVTLDTSKKSSSLLITASRAADTASYFCATDGNQFYFGTGTSLTVIP and β GAVVSQHPSRVICKSGTSVKIECRSLDFQATTMFWYRQFPKQSLMLMATSNEGSKATYEQGVEKDKFLINHASLTLSTLTVTSAHPEDSSFYICSARGQGENVYGYTFGSGTRLTVV, alignment against the model PDB 2IJ0 [40], and the deposited HLA classII, DRB10 sequence UniProtKB Q30167 [63]. The final model was then created by superposition of the TCR, TCRβ from residues 0 to 244 and the TCRα residues 1 to 190, onto the HLA peptide, truncated at the first residue on the extracellular membrane side, by root mean square alignment to model PDB ID 1FYT [64]. The SMX structure was generated using chemsketch [65], converted to PDB format with openbabel and docked into the TCR using Autodock and Autodock Vina [66,67], showing a strong affinity at a site in the CDR2β in prior computational studies. This initial docking study utilized 60 rigid and 60 flexible global dockings of SMX against multiple models of TCR, and a TCR containing Vβ20-1 chosen because of an SMX docked site free of the pHLA interface. Each TCR model was prior to docking energy minimized, and equilibrated in SPC water at 300K. For the TCR used here, a refined set of 60 flexible docking runs were conducted where amino acids defining the CDR2β, res 54-71, were allowed movement. For these the docking site was limited to the CDR2β loop dimensions. The highest affinity docked conformation of SMX was then used in initial SMX bound models. The program PRODGR was initially used to generate an all atom topology for the SMX molecule [68], and minor changes to the charge set corrected from the IUPAC standards (www.IUPAC.org). This topology was used in all simulations.

### Peptide Ligand Determination

Energy minimization of the initial structure from Swiss-modeller was done in Gromacs [69], using only SPC water and a 53a6 force field [70] at a temperature of 300K. After several preliminary large scale screening processes, a working computational method was developed to screen small peptide fragments. From this, a 15 amino acid peptide sequence, GATGRKCDGCKHWHA corresponding to human Lamininα2, was determined using a modified AutoGrow [71] script (http://sourceforge.net/p/autmatedpeptide) to generate random peptide fragments, and docking these using Autodock Vina [66], to the TCR-HLA structure. A complete screening method was to define two separate docking areas beneath the CDR3 loops and encompassing half the HLA peptide binding pocket. These areas overlap for 4-5Å to accommodate a single end amino acid in any resulting peptides between docking sites, which lie on top of each other in the mid portion of the HLA peptide groove. Either defined area was large enough to allow 4-5 linear amino acids to fit within the dimensions based on initial starting TCR-HLA models. Software was then given a linear 4-5 amino acid peptide, and allowed to mutate these to form a library of non-redundant peptide fragments. Both CDR3 loops were allowed complete amino acid flexibility, including the backbone bonds, and each defined area docked in separate batch runs. A series of 5 runs, per 10 cycles, at 100 screened mutations per cycle allowed for the determination of a high affinity set of 2-3 ligands from either defined area. Approximately 7500 fragments were screened in this way. From these, several 7-8 amino acid chimeras were produced from overlapped amino acids at the HLA mid groove portion and protein-blast (NCBI/NIH ref) used to determine the suspected proteins used as a self-ligand. Using the protein sequences retrieved, peptides of 9-15 amino acids were chosen based on sequence alignments over the HLA binding groove and the docked small fragments corresponding to the identified proteins. These resulting peptides were docked into the whole structure using Autodock Vina, retained flexibility in the CDR3 loops, and allowing half of the ratable backbone bonds of the peptide to rotate. Starting at 8 amino acid fragments and moving to 15 amino fragments, 1 residue was added for each generated peptide based on the identified protein amino sequence, giving 8 different length proteins overall. Allowing all peptide backbone bonds to rotate prevented any docked conformations to be determined, often resulting in circular or globular peptide orientations. Repeated docking is necessary for each defined larger peptide fragment, as the conformational space for each ligand increases with size of the peptide, and at least a dozen docking runs conducted per peptide needed to compare fragments. Affinity based on averaging all docking runs individual calculated affinities was used to determine the final best ligand.

### Initial Molecular Dynamic Model Equilibration

From the complete structure with bound peptide, solvation with SPC H2O, NaCl, and KCl at 0.15 and 0.08 M, pH 7.4 through amino protination state, were performed in Gromacs using the 53a6 Gromos force field, and equilibrated over several ns at 300K temperature, 1ATM pressure using a Nose-Hoover thermostat [72,73] and Parrinello-Rahman pressure coupling. Initial MD simulations were set using a TCR with bound, and unbound SMX. For both of these, solvent was 40,000 SPC water, 48 K+, 129 Na+, and 177 Cl- atoms, in a simulation box of 90.122, 164.222 and 90.122 Å. A 4 ns, unconstrained MD for the SMX, or non-SMX simulations were then conducted, and initial structures taken every 364 ps for production run pulled simulations.

### Production Run MD Simulations

Initial steered molecular dynamics were then set as 11 with SMX and 10 without SMX pull simulations using starting structures spaced as described from the un-restrained 4 ns simulation as starting points, and a modified protocol for TCR-pHLA free energy calculations described previously [37]. Pull force was set as a range for each run from start 0 to finish 2000 kJ/mol*nm^2^, as determined maximal force for the TCR with SMX found in trial runs, to completely separate the TCR from the pHLA. Pull force ranges were kept constant for without SMX simulations to reduce overall error estimates in energy comparisons between the two sets of trajectories. This force was applied harmonically in increments to the COM of the TCR in both simulation sets, along the Z axis of the simulation (Figure 1). A pull distance was set at 0.0005 nm/ps, for a total of 4ns each simulation along the Z axis. In each simulation, the pHLA was positionally restrained. Bond constraints were kept constant using LINCS. A distance of 20 Å was achieved for each simulation between the TCR and pHLA, with a mean complete separation time of all long range interactions between the TCR and pHLA at 3.2 ns. Center of mass, and rotational effects were removed from overall simulations by linear correlation COM removal set in the initial parameters for the entire system every 2 fs intervals, and 300K temperature, 1ATM pressure kept constant using a Nose-Hoover thermostat and Parrinello-Rahman pressure coupling. All short-range interactions were truncated at 9Å, temperature coupling conducted at 0.1 ps, and pressure coupling at 1 ps. Wall forces were not used as described in the published protocol, as box size was increased in the X and Y dimensions.

### Data Analysis

Analysis was performed using Gromacs, and auxiliary software from numpy [74], Pymol (© Pymol Mol.Graphics Schrödinger, LLC) and VMD [75], linux gnumeric (GPL license) and qtiplot/SciDavis. For analysis, statistics were determined from total energies taken at 2ps frames for total ΔG [76] using pull force, and 20ps frames for all other energy calculations. All trajectories were treated as separate, and averaged for particular data involved respective of the two separate sets of simulations. Fitting of structures was to initial starting structures for the respective trajectory, and either Cα or all atoms used as indicated. RMSD and RMSF were calculated in gromacs [47] using internal tools, using all atoms. PCA and covariance [43,77] were calculated from raw trajectories and energy files using g_anaeig and g_covar tools in Gromacs against each trajectories starting model, converted to matrices using a set of matrix manipulation scripts written in numpy, and averaged in qtiplot/SciDavis for figures to represent mean distributions of energies. Figures were generated in qtiplot/SciDavis.

Analysis of short-range interactions (Figure 4) were conducted in two parts. For specific variable domains, all Leonard-Jones and Coulomb short-range interactions were summed for self and pHLA at a 9 Å cut off. All interactions with neighboring domains, or solvent were excluded. Self-interactions were defined as atom-atom interactions within the defined index groups, excluding other TCR atoms. Index groups analyzed were all atoms respectively in residues α40-69, CDR1-2α, α84-94, CDR3α, β46-76, CDR1-2β, and β94-106, CDR3β (Figure 4A). For the whole TCR (Figure 4B), all atoms within the TCR were included, and all other atoms in the model including pHLA were excluded. Energies were extracted from energy files, and zeroed by hand for each trajectory. These were then aligned and standard deviation and means across all runs calculated using qtiplot/SciDavis.

For PCA positional mapping, the initial structure using all TCR or TCR domain atoms for each trajectory was projected onto the corresponding vectors, and positions determined for each atom on the plot as X an Y ordinates by hand. An additional set of indexed atoms included α25-39, CDR1-2α bridge and β32-45, CDR1-2β bridge. PCA filtered trajectories were visually inspected in VMD and pymol rendered movies to determine vector direction. Additional filtered trajectories using gromacs tools g_filter to remove random fluctuations and used for movie S6, S7 with filter set at 3. Hydrogen bonding was determined in gromacs. Distances were determined in gromacs using built in software analytical tools for individual trajectories, and average and error estimates calculated in qtiplot/SciDavis. Pearson’s linear correlation values were calculated in gnumeric using each vector mean against distance mean. All other analysis was conducted using Gromacs internal scripts, internal software analytical tools, or software stated in combination. The initial Gromacs used was 4.5.2 for all simulations, however 4.5.5 was used for analysis.

## Supporting Information

Figure S1 **Free Energy Changes of Simulations.** Free energy change for individual trajectories **A**) with SMX, **B**) without SMX. In A,B trajectory number indicates time point from initial run taken evenly spaced as indicated in methods. **C**) Wham analysis of same trajectories as shown in Figure 3, for comparison. These incorporate autocorrelation functions, and bootstrap analysis. The original experimental design incorporated a lambda value but was not used in single histogram analysis shown in Figure 3.
(TIFF)

Figure S2 **Center of Mass Definitions.** Center of mass (COM) shown in Figure 4. Centers are colored balls, CDR2β, blue, CDR3β, pink, CDR3α yellow (hidden under loop), and CDR2α light blue. Lines are 6 different measured distances shown in graphs 1 and 2, from Figure 6 B. Centers were drawn as close as possible to calculated COM, using pymol, and the closest atoms to the calculated point. These may be off by 1-2 Å, and are for reference to Figure 6 only.
(TIFF)

Figure S3 **Positionally Mapped Domains and Residues.** **A**) Thick lines purple Jβ, blue Jα. These are more equally distributed without SMX. Thin lines, red, CDR1-2α loops, black, CDR3α, green, CDR1-2β loops, orange, CDR3β, pink, CDR1-2α spanning loop, maroon, CDR1-2β spanning loop. **B**) **With SMX** 1, CD1-2α spanning loop, 2, Top CDR1α, 3, CDR3α, 4, CDR3α peptide contact, 5, Constant β, 6, Constant α, 7, CDR2-3α spanning loop, 8, CDR2β bottom, 9, CDR1α bottom, 10, Constant β domain, 11, Constant α to Jα contact, 12, ASP64β, 13, LYS 65β, 14, CD2β bottom, 15, CDR1-2α spanning loop. **Without SMX** 1, CDR1-2α spanning loop, 2, CDR3α, 3, CDR1β to Jβ interaction, 4,5, CDR3β, 6, Constant β, 7, CDR2-3α spanning loop, 8, Constant α, 9, Constant β, 10, Jβ, 11, CDR2β bottom, 12, CDR1α bottom, 13, CDR1α mid, 14, CDR1α upper loop, 15, CDR2-3β spanning loop, 16, Jα, 17 CDR2β. **C**) Peaks are residue specific. Without SMX a 15 amino acid loop spanning the constant domain from the β to α is stopped by CDRβ loops forcing VALα143 inward through hydrophobic interactions. With SMX a spring like movement to the top of the α loops results in PHEα158 moving. In figure CD refers to CDR loop.
(TIFF)

Figure S4 **Remaining Sets of PCA Analyzed for TCR Domain Energy and Motion Determination.** Remainder of all energy projections from PCA analysis for varied domains analyzed on the TCR. A summary of the principal motions corresponding to these first 3 vectors is shown in Figure 8, A) and **B**) for SMX and no SMX models. All PC vs PC, indicated on axis, are shown as with, and without SMX directly below. Energy changes can be correlated to principal motions, however only those indicated in the paper text are in the same directions, regarding vector motions, indicated in Figure 7. Note energy levels also vary for some heat maps shown significantly. This is highlighted by CDR3β, which also shows a larger degree of scattered low energies as a result.
(TIFF)

Figure S5 **Additional Ramachandran Plots.** Ramachandran plots of other residues in the hydrogen bonding network affected by SMX indirectly. These show several features; For ARG 36 on the CDR1-2β spanning loop, the only observable difference is a very small increase in degree of movement around a set angle, even though the residue moves 3-4 Å in the bound SMX Vs. unbound simulations. Residue GLU 62 however represents a change between bound TCR with or without SMX, while the free TCR in either is the same. GLU86 and ASP 87 both show complete conformational changes of the residue positions throughout the trajectories between the two simulations, with a minor degree of overlap. Together these show that Ramachandran angle analysis can highlight residue changes, however a structural difference may be missed if the angles are the same, even if the residues overall position is significantly different. Intensity, right color graph, indicates percent occupancy across all trajectories. Top plot, with SMX, bottom, without.
(TIFF)

Figure S6 **Residues shown in Movie S8 as stick models.**
(TIFF)

Figure S7 **Residues shown in Movie S9 as stick models, these are the same residues as Figure S6.**
(TIFF)

Movie S1 **Example 4 Nanosecond Simulation With SMX.** Simulation with SMX for trajectory 5. This shows a single MD simulation as a representation of the entire MD analyzed for each trajectory set overall with SMX present. All atoms are shown as wire models. The pHLA is held positionally restrained, however amino acid side chains have free movement.
(AVI)

Movie S2 **Example 4 Nanosecond Simulation Without SMX.** Simulation without SMX for trajectory trajn_5. These show single MD simulations as a representation of the entire MD analyzed for each trajectory set overall. All atoms are shown as wire models. The pHLA is held positionally restrained, however amino acid side chains have free movement.
(AVI)

Movie S3 **Principle Component 1 Primary Motion.** Movie of Vector 1, or Principal component 1 (PC1). The trajectory was filtered using Gromacs tools to only show movements along the primary eigenvector produced from covariance analysis and using the TCR coordinates. Vector analysis produces vectors ordered according to descending overall contribution to the total energy found in the MD simulations for the analyzed protein or portion of protein involved, which are also plotted energy wise in Figure 5,6 and Figure S6. The first 3 vectors produced represent 90, or 90.1% of all energy and overall motion.
(AVI)

Movie S4 **Principle Component 2 Primary Motion.** Movie of Vector 2, or Principal component 2 (PC2). The trajectory was filtered using Gromacs tools to only show movements along the secondary eigenvector produced from covariance analysis and using the TCR coordinates.
(AVI)

Movie S5 **Principle Component 3 Primary Motion.** Movie of Vector 3, or Principal component 3 (PC3). The trajectory was filtered using Gromacs tools to only show movements along the tertiary eigenvector produced from covariance analysis and using the TCR coordinates.
(AVI)

Movie S6 **Filtered Random Motion Removal for Example SMX Containing Trajectory.** Filtered trajectory for simulation shown in movie 1. Filtered trajectories use averaging of motion with N=3 using the Gromacs filter (cosine(π*time frames/N) to remove random movements caused by oscillation or random solvent effects. Resultant motions are non-random and associated with overall domain changes in the TCR as it moves from pHLA bound to free in solvent across the simulation. These motions are also observed from PCA analysis and simple RMSD or RMSF analysis in static representations. Trajectory 5 was used to produce movie.
(AVI)

Movie S7 **Filtered Random Motion Removal for Example Trajectory.** Filtered trajectory for simulation shown in movie 2. Filtered trajectories use averaging of motion with N=3 using the Gromacs filter (cosine(π*time frames/N) to remove random movements caused by oscillation or random solvent effects. Resultant motions are non-random and associated with overall domain changes in the TCR as it moves from pHLA bound to free in solvent across the simulation. These motions are also observed from PCA analysis and simple RMSD or RMSF analysis in static representations. Trajectory n5 was used to produce movie.
(AVI)

Movie S8 **Example Trajectories Showing Important Residues in CDR2β Switch With SMX.** SMX effects on the Vβ20-1 domain. Residues shown as sticks are labeled in the static image, Figure S6. Differences are observed in the initial structures between Movies S8 and S9. Overall, two amino acids, ASP 64 and LYS 65 effect a larger translated hydrogen bonding along the outer portions of the separate β loops, bridging between loops. The long range effects shown are seen to culminate in the constant loop region from β220-230, and also the position of the distal end of the CDR1-2β spanning loop. This shows how two single hydrogen bonds can be amplified and transmit a much larger signal across a protein from a ligand. SMX shown as wire model.
(AVI)

Movie S9 **Example Trajectories Showing Important Residues in CDR2β Switch Without SMX.** The Vβ20-1 domain without SMX. Residues shown as sticks are labeled in the static image Figures S6 and S7. Differences are observed in the initial structures, and the entire pHLA dissociation can be compared to see how long range differences are transmitted on the outer surface of the TCR. Cover of movie 9, Figure S7, are same residues labeled in Figure S6, held in different positions without SMX.
(AVI)

Table S1 **Standard error for all distance measurements shown in Figure 5, listed as individual atom pairs.** Atoms used vary between residue and are reflected in increases distance from Cα, as shown by residue β65 (LYS terminal N) Vs. α46 (GLU back bone O) in standard error averaged across all trajectories.
(XLS)

Table S2 **Pearsons linear correlation coefficient calculated for atom linear movement against the first three PC of the entire TCR.** Labels are atom pair and V, vector used, followed by calculated Pearsons r-value from mean averaged linear distances from all trajectories. These indicate differences in correlated motions, between SMX or no SMX TCR, and are the same atom pairs shown in Figure 6B.
(XLS)

Table S3 **Selected hydrogen bonds found by tabulating all trajectories, with or without SMX.** All hydrogen bonds are shown for TCR and HLA or TCR and peptide, with number of total hydrogen bonds shown. These bonds oscillate and as shown in Figure S6, only 15 without and 12 with on average are present per the first 200-300 picoseconds. Select hydrogen bonds for loops in the Vα, Alpha loops, or Vβ, Beta loops, Beta 1-2 bridge, and Alpha 1-2 Bridge refer to loops connecting the CDR1 and CDR2 loops, and are upper facing the constant domain of the TCR. For these hydrogen bonds between other residues in the TCR are listed, based on possible correlation with TCR structural change, and selected based on visual inspection of starting structure as a means to check suspected changes. Not all bonds found in the TCR are listed. **, bonds which show high degree of oscillation between frames and trajectories. BB, back bone interaction. Not all backbone interactions are indicated.
(XLS)
