# Supplementary figures and images for: Sulfamethoxazole Induces a Switch Mechanism in T Cell Receptors Containing TCRVβ20-1, Altering pHLA Recognition
Source: PLoS One. 2013 Oct 7;8(10):e76211. doi: 10.1371/journal.pone.0076211 (PMC3792127; doi:10.1371/journal.pone.0076211)

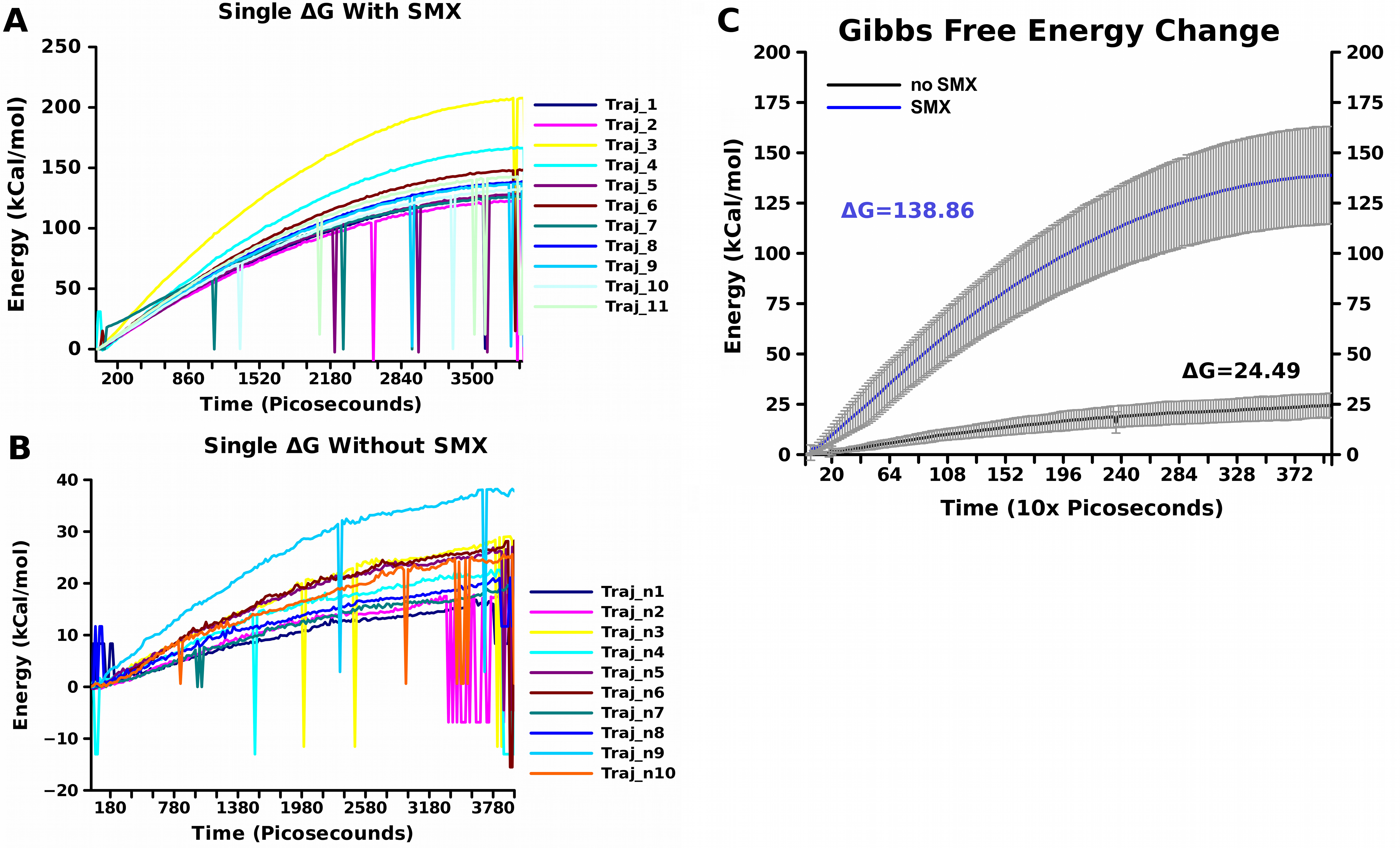

Supplement: Figure S1 — Free Energy Changes of Simulations. Free energy change for individual trajectories A) with SMX, B) without SMX. In A,B trajectory number indicates time point from initial run taken evenly spaced as indicated in methods. C) Wham analysis of same trajectories as shown in Figure 3, for comparison. These incorporate autocorrelation functions, and bootstrap analysis. The original experimental design incorporated a lambda value but was not used in single histogram analysis shown in Figure 3. (TIFF) [file pone.0076211.s001.tiff]

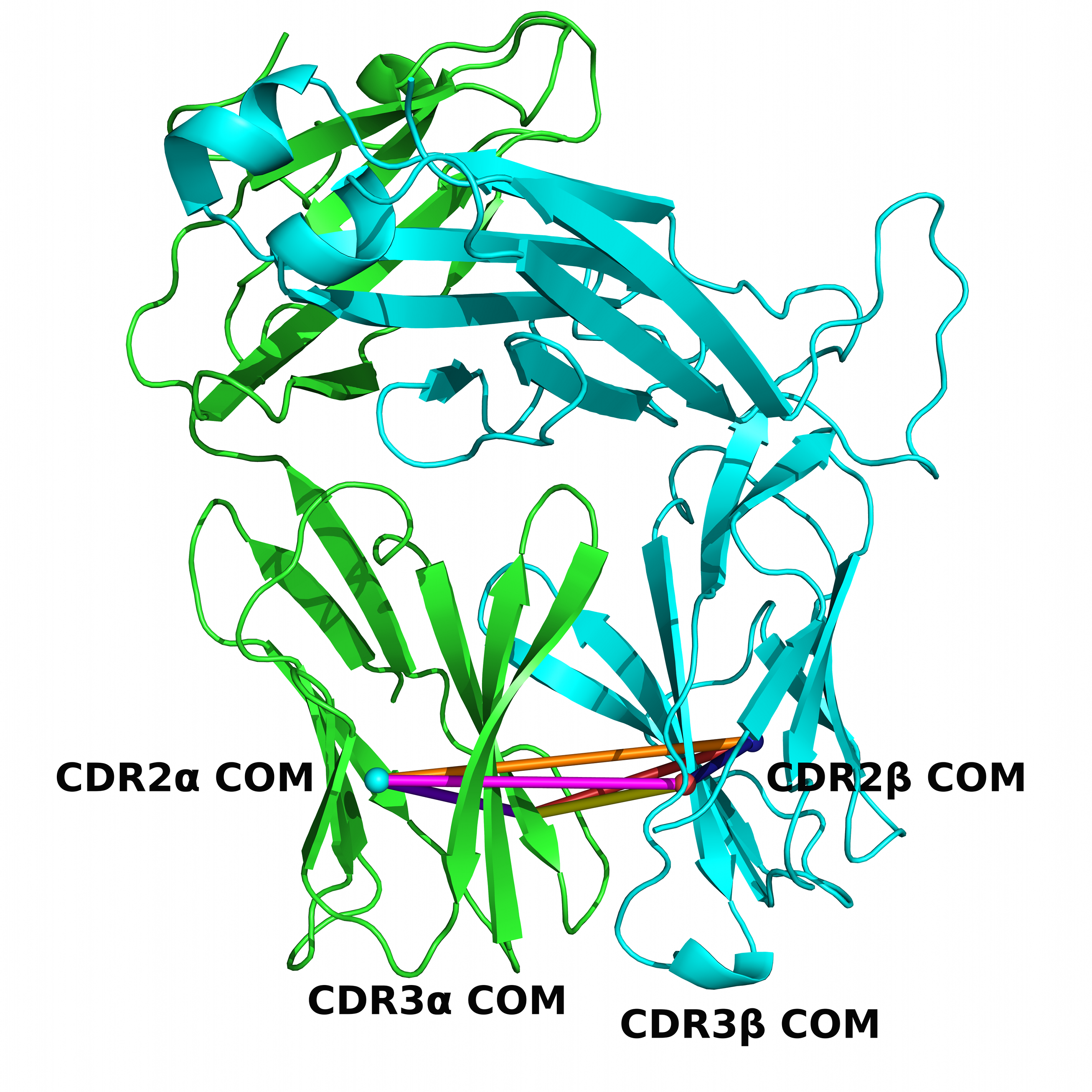

Supplement: Figure S2 — Center of Mass Definitions. Center of mass (COM) shown in Figure 4. Centers are colored balls, CDR2β, blue, CDR3β, pink, CDR3α yellow (hidden under loop), and CDR2α light blue. Lines are 6 different measured distances shown in graphs 1 and 2, from Figure 6 B. Centers were drawn as close as possible to calculated COM, using pymol, and the closest atoms to the calculated point. These may be off by 1-2 Å, and are for reference to Figure 6 only. (TIFF) [file pone.0076211.s002.tiff]

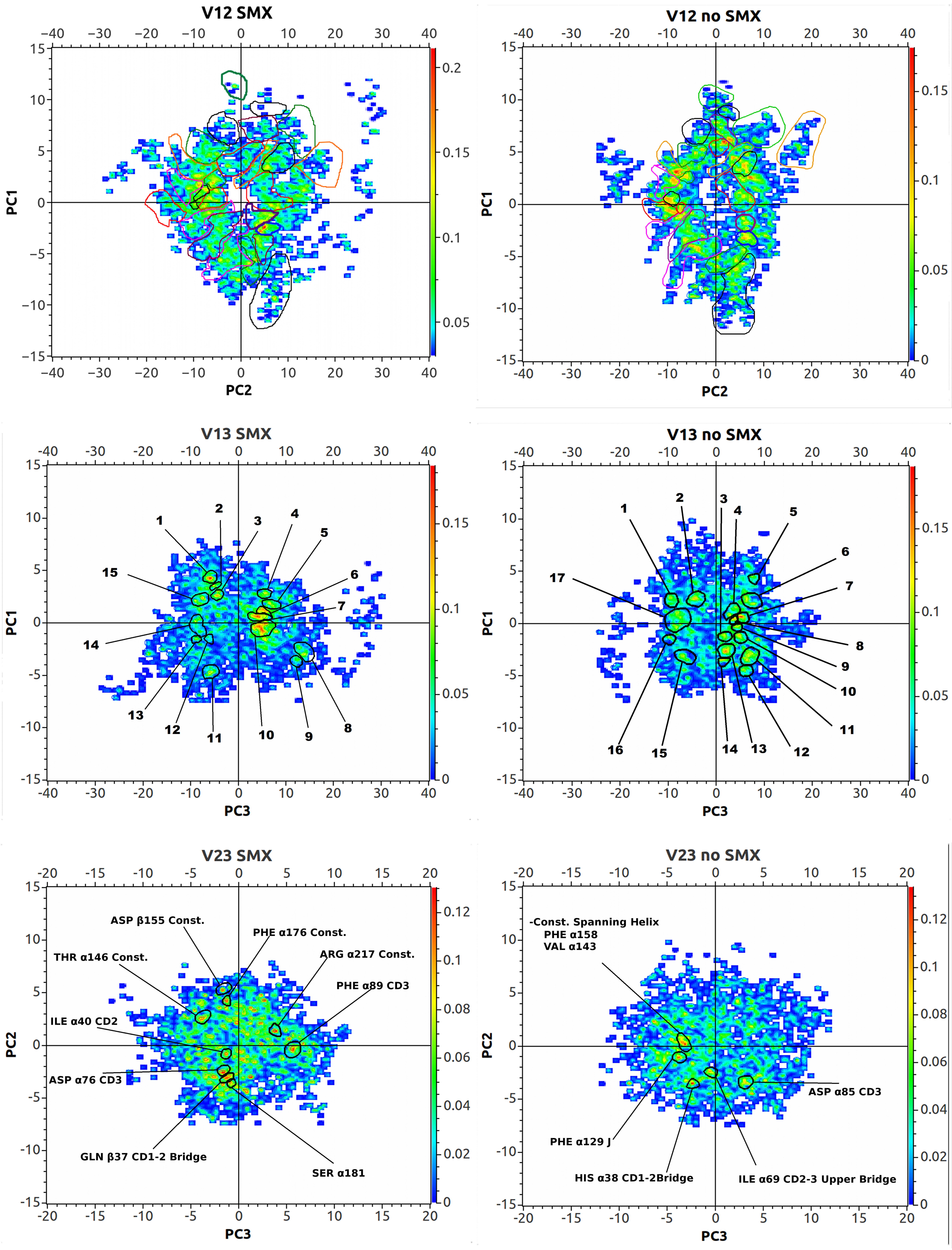

Supplement: Figure S3 — Positionally Mapped Domains and Residues. A) Thick lines purple Jβ, blue Jα. These are more equally distributed without SMX. Thin lines, red, CDR1-2α loops, black, CDR3α, green, CDR1-2β loops, orange, CDR3β, pink, CDR1-2α spanning loop, maroon, CDR1-2β spanning loop. B) With SMX 1, CD1-2α spanning loop, 2, Top CDR1α, 3, CDR3α, 4, CDR3α peptide contact, 5, Constant β, 6, Constant α, 7, CDR2-3α spanning loop, 8, CDR2β bottom, 9, CDR1α bottom, 10, Constant β domain, 11, Constant α to Jα contact, 12, ASP64β, 13, LYS 65β, 14, CD2β bottom, 15, CDR1-2α spanning loop. Without SMX 1, CDR1-2α spanning loop, 2, CDR3α, 3, CDR1β to Jβ interaction, 4,5, CDR3β, 6, Constant β, 7, CDR2-3α spanning loop, 8, Constant α, 9, Constant β, 10, Jβ, 11, CDR2β bottom, 12, CDR1α bottom, 13, CDR1α mid, 14, CDR1α upper loop, 15, CDR2-3β spanning loop, 16, Jα, 17 CDR2β. C) Peaks are residue specific. Without SMX a 15 amino acid loop spanning the constant domain from the β to α is stopped by CDRβ loops forcing VALα143 inward through hydrophobic interactions. With SMX a spring like movement to the top of the α loops results in PHEα158 moving. In figure CD refers to CDR loop. (TIFF) [file pone.0076211.s003.tiff]

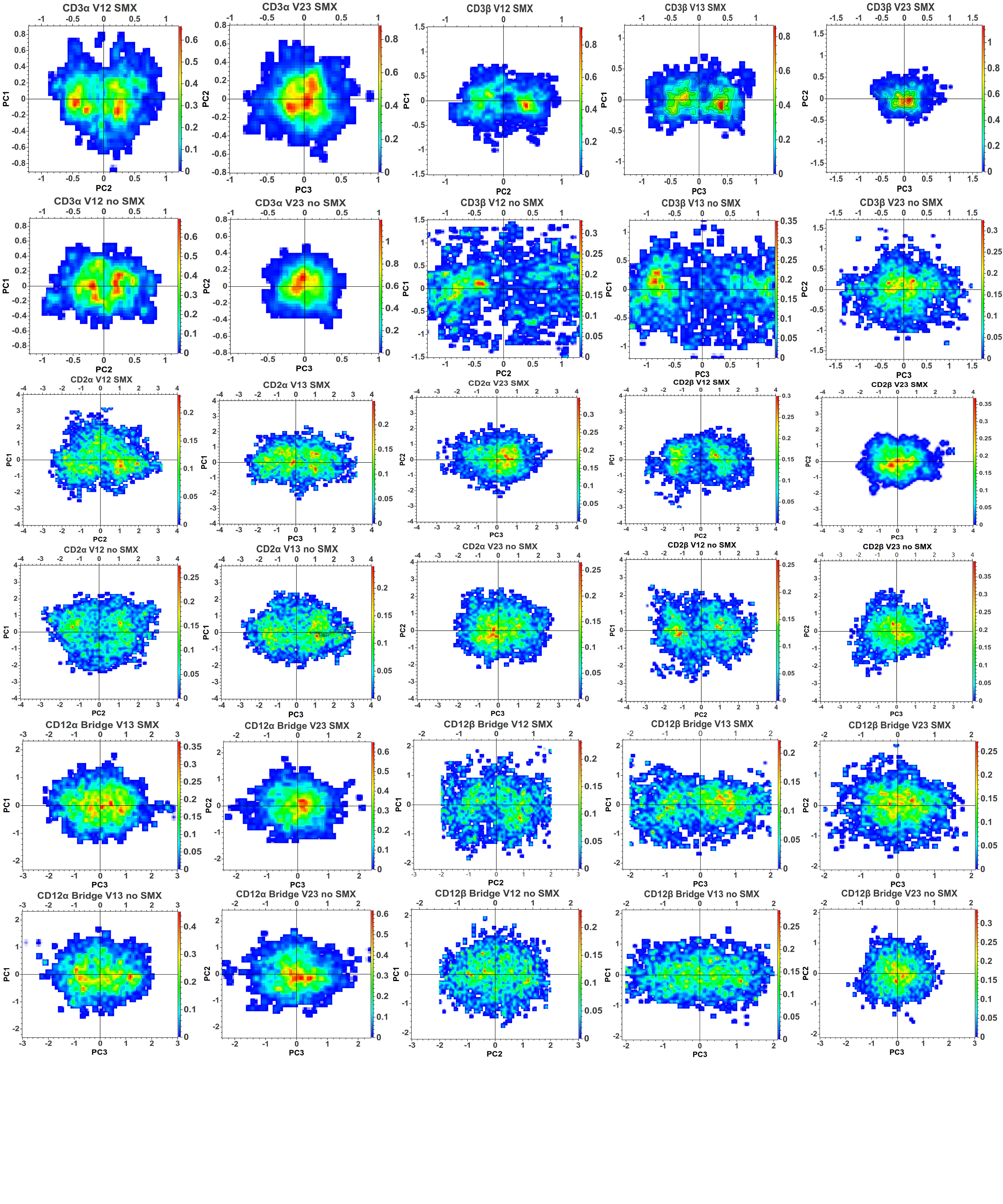

Supplement: Figure S4 — Remaining Sets of PCA Analyzed for TCR Domain Energy and Motion Determination. Remainder of all energy projections from PCA analysis for varied domains analyzed on the TCR. A summary of the principal motions corresponding to these first 3 vectors is shown in Figure 8, A) and B) for SMX and no SMX models. All PC vs PC, indicated on axis, are shown as with, and without SMX directly below. Energy changes can be correlated to principal motions, however only those indicated in the paper text are in the same directions, regarding vector motions, indicated in Figure 7. Note energy levels also vary for some heat maps shown significantly. This is highlighted by CDR3β, which also shows a larger degree of scattered low energies as a result. (TIFF) [file pone.0076211.s004.tiff]

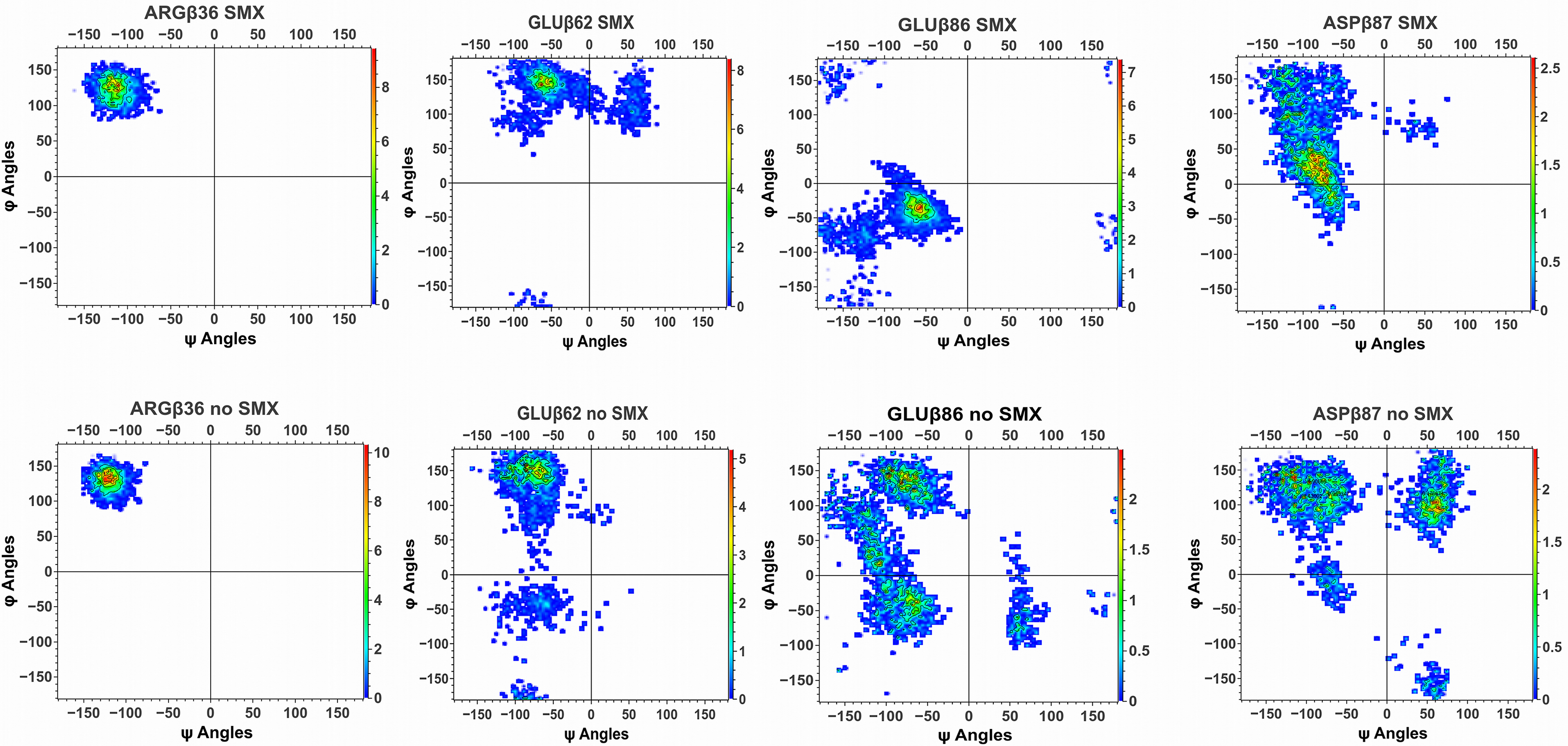

Supplement: Figure S5 — Additional Ramachandran Plots. Ramachandran plots of other residues in the hydrogen bonding network affected by SMX indirectly. These show several features; For ARG 36 on the CDR1-2β spanning loop, the only observable difference is a very small increase in degree of movement around a set angle, even though the residue moves 3-4 Å in the bound SMX Vs. unbound simulations. Residue GLU 62 however represents a change between bound TCR with or without SMX, while the free TCR in either is the same. GLU86 and ASP 87 both show complete conformational changes of the residue positions throughout the trajectories between the two simulations, with a minor degree of overlap. Together these show that Ramachandran angle analysis can highlight residue changes, however a structural difference may be missed if the angles are the same, even if the residues overall position is significantly different. Intensity, right color graph, indicates percent occupancy across all trajectories. Top plot, with SMX, bottom, without. (TIFF) [file pone.0076211.s005.tiff]

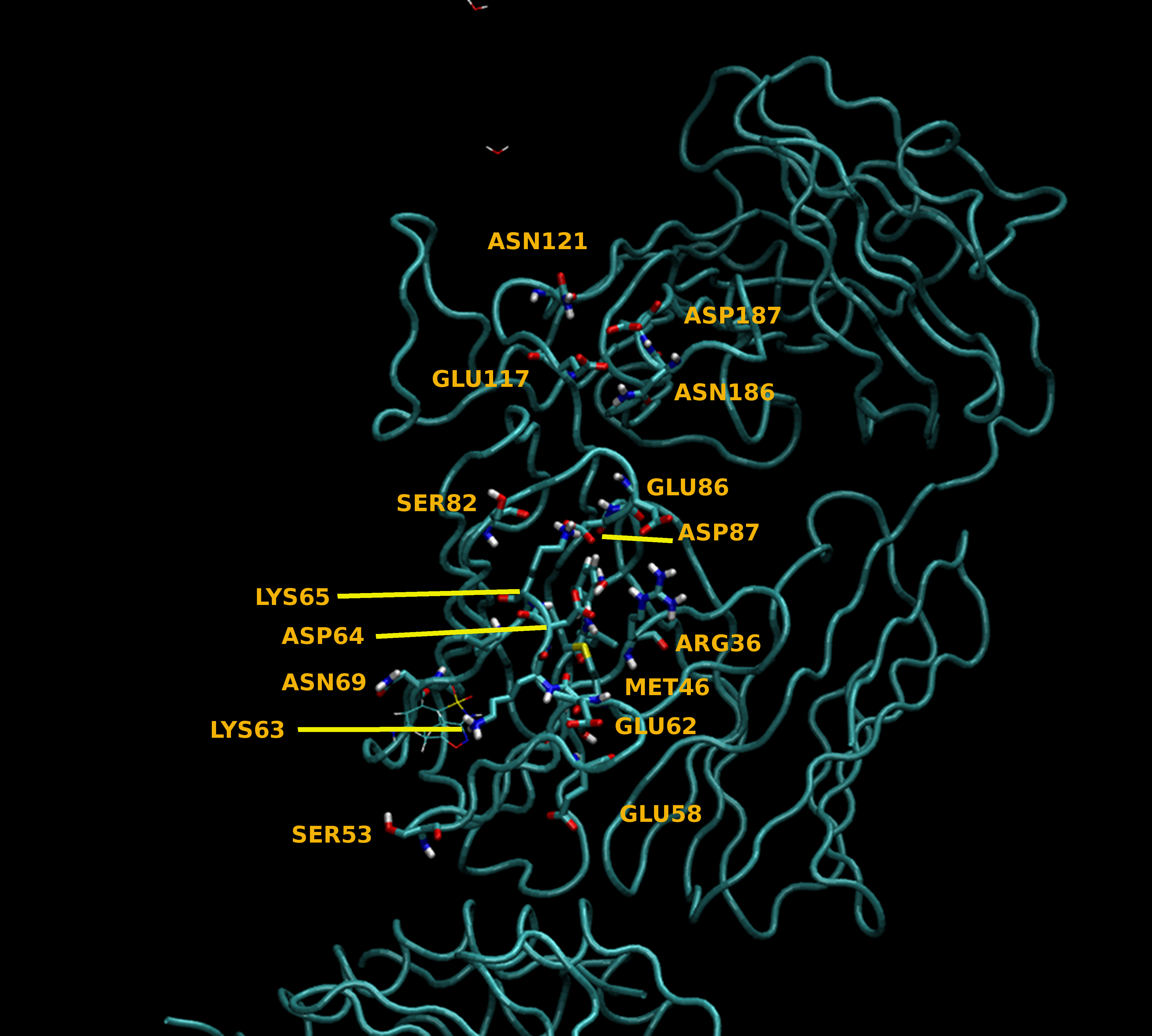

Supplement: Figure S6 — Residues shown in Movie S8 as stick models. (TIFF) [file pone.0076211.s006.tiff]

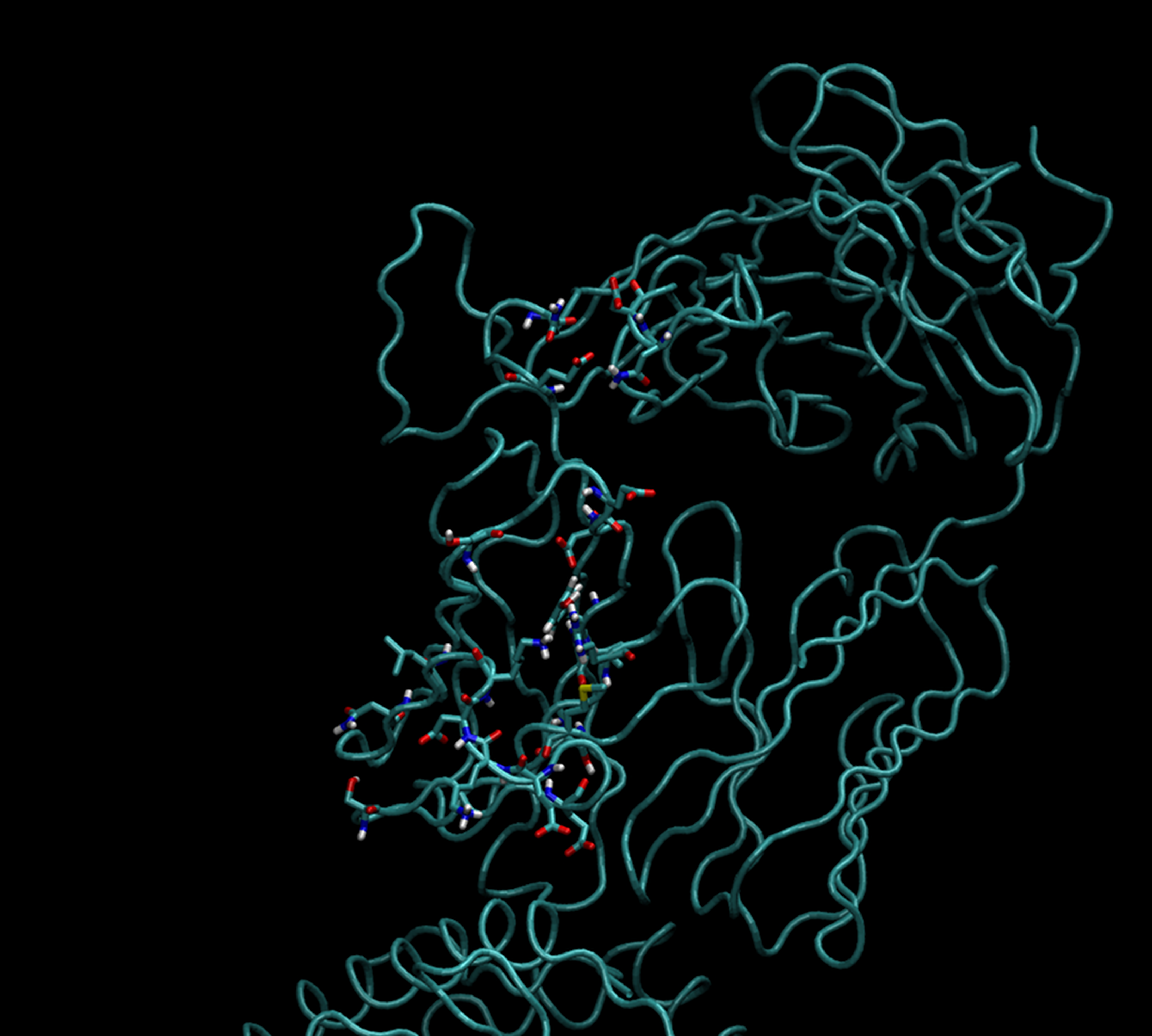

Supplement: Figure S7 — Residues shown in Movie S9 as stick models, these are the same residues as Figure S6. (TIFF) [file pone.0076211.s007.tiff]
